# Epitope-based vaccine of NiV targeting glycoprotein and fusion protein: an integrated immunoinformatics and bioinformatics approach

**DOI:** 10.1016/j.virusres.2026.199746

**Published:** 2026-05-15

**Authors:** Waheeb Sami Aggad, Saurav Kumar Mishra, Taha Alqahtani, Abdelkrim Guendouzi, Zsolt Tóth, Magdi E.A. Zaki

**Affiliations:** a Division of Anatomy, Department of Basic Medical Sciences, College of Medicine, University of Jeddah, Jeddah, 23890, Saudi Arabia; b Department of Bioinformatics, University of North Bengal, District-Darjeeling, West Bengal 734013, India; c Département of Pharmacology, College of Pharmacy, King Khalid University, Abha 62529, Saudi Arabia; d Laboratory of Chemistry: Synthesis, Properties and Application, Faculty of Sciences and Technology, University of Saida, Saïda, Algeria; e Faculty of Wood Engineering and Creative Industries, University of Sopron, Hungary; f Department of Chemistry, College of Science, Imam Mohammad Ibn Saud Islamic University (IMSIU), 11623 Riyadh, Saudi Arabia

**Keywords:** Nipah virus, Bioinformatics, Epitope, Vaccine, Simulation, Immune response

## Abstract

NiV is an ongoing infection that causes severe complications, and its frequent outbreaks make its treatment more complicated in terms of the proper therapeutic design. No viable vaccine candidate exists to combat NiV; however, efforts are underway, and some vaccines are in clinical trials. Therefore, an integrated immunoinformatics and bioinformatics approach was employed to design a promising epitope-based vaccine targeting glycoprotein and fusion protein, which are critical for host–pathogen interactions. The two targets, i.e., glycoprotein and fusion protein, were examined to identify B- and T-cell epitopes. A total of 3 B cell, 13 MHC I and 7 MHC II epitopes from both targets were chosen for vaccine formulation. Epitopes were joined using specific linkers and combined with three different adjuvants to generate 3 vaccines, i.e., VC1, VC2, and VC3, enabling selection of the best construct based on antigenicity. PADRE and a 6 × His-tag were also included. Analysis resulted in VC2 as a highly immunodominant profile. The incorporated epitopes resulted in broad global population coverage ( 98.97%) and remarkable stereochemical properties of the modelled vaccine, as assessed via 2D and 3D analyses. Subsequently, the VC2 were docked to the toll-like receptors (TLR2, TLR3, and TLR4) and showed high binding affinities of -14.2 to -23.1kcal/mol, along with H-bonds (9 to 20). Among them, the VC2–TLR3 complex showed the most promising interaction and demonstrated stability based on the NMA analysis (eigenvalue = 1.710065e-06), followed by a 100-ns simulation and RMSD, RMSF, etc. Furthermore, the immune simulation and expression analysis revealed remarkable immune activity in a dose-dependent manner, yielding promising GC% and CAI values. Based on the integrated framework, the study demonstrated that the designed vaccine, incorporating variations in the adjuvant, may exhibit significant immunodominant activity and could help combat NiV.

## Highlights

• Glycoprotein and fusion proteins were systematically analyzed to identify immunogenic epitopes for rational vaccine design.
• 3 vaccine constructs were formulated, and VC2 was prioritized based on the variability and incorporation of different adjuvants.
• The formulated vaccine constructs exhibited strong immunodominant features.
• Docking analysis demonstrated favorable interaction of the vaccine with TLR3 compared to other TLRs, and the complexes were further evaluated for stability using Normal Mode Analysis (NMA) and 100 ns molecular dynamics simulation.
• Dose-dependent immune simulation demonstrated robust immune activity, and GC content analysis suggested high expression potential of the construct.

## 1 Introduction

Nipah virus (NiV) is an emerging virus that can cause severe infections in individuals. Its outbreaks have been associated with high fatality rates and can be severe and complicated. It is a highly pathogenic RNA virus with a negative-sense genome from the zoonotic viruses of the *Henipavirus* (genus), *Paramyxoviridae* (family) (Banerjee et al., 2019; Shabbir et al., 2025; Wang et al., 2001). Moreover, the first case of NiV was reported in Malaysia in 1998, after which it spread to other regions, including India, Bangladesh, and the Philippines (Chua et al., 2000; Sharma et al., 2025). Based on epidemiological characteristics, NiV diversity was observed across different countries, followed by variation in transmission patterns. Among variations and infection cases, Bangladesh records the highest incidence (Khan et al., 2024; Sharma et al., 2025). The symptoms include muscle pain, dizziness, etc., and the severity of the infection can lead to encephalitis, respiratory complications, and other complications (Sharma et al., 2025). The genome of NiV is encoded by different genes, such as attachment glycoprotein (G) and fusion glycoprotein, facilitating binding and viral entry in host cells, nucleocapsid protein (N) vital in replication and transcription, phosphoprotein (P) crucial for viral RNA synthesis, matrix (M) vital in viral assembly, and lastly long polymerase (L) viral RNA replication (Banico et al., 2024; Oladipo et al., 2025; Yang et al., 2024). Among the other targets, the attachment glycoprotein (G) and fusion glycoprotein were identified as more ideal targets for vaccine design based on pathogenesis, such as vital in host-pathogen interactions following binding, fusion, and viral entry (Loomis et al., 2021; Salleh, 2025). Currently, no effective vaccine is available to combat NiV; however, efforts are underway, and some vaccines are in preclinical stage development, formulated using different strategy (Leyva-Grado et al., 2024). Regarding vaccine formulation, researchers found that a rational strategy is more advantageous than a conventional one in terms of precision targeting, rapid development, and cost-effectiveness (Fadaka et al., 2021; Oli et al., 2020). Utilising immunoinformatics, along with *in silico* steps, was found to be promising for developing and constructing an epitope-based vaccine, considering the highly promising B- and T-cell epitopes' ability to recognise antigenic components, which further trigger the immune response to combat the infection. Concerning NiV using the immunoinformatics, Bioinformatics and reverse vaccinology approach, the researchers made an effort towards the vaccine formulation (Banico et al., 2024; Kaur et al., 2024; Kim et al., 2025; Rahman et al., 2022; Sharma et al., 2025); however, prioritizing the vaccine based on its immunogenic profile following the highly potent epitope selection, **c**omparative evaluation of different adjuvant systems, and integrated immunogenicity-based ranking of vaccine constructs along with the priorization of framework based on the antigenicity filtering strategies remain underexplored which may help achieve a significant outcome in vaccine design to combat NiV and reduce infection in individuals. Therefore, this study aims to formulate a potent epitope-based vaccine targeting the NiV glycoprotein and fusion protein by integrating immunoinformatics and bioinformatics approaches. Three vaccines based on the adjuvant were designed, and their immunological and essential features were examined accordingly. The vaccine's activity towards the TLRs (TLR2, TLR3, and TLR4) was assessed via docking, and stability was analysed via simulation. The immune activity of the prophylactic vaccine among the designed vaccines was examined over time. The designed prophylactic vaccine among the formulated ones showed a significant profile and may trigger an immune response, helping combat NiV.

## 2 Materials and methods

**Fig. 1:**
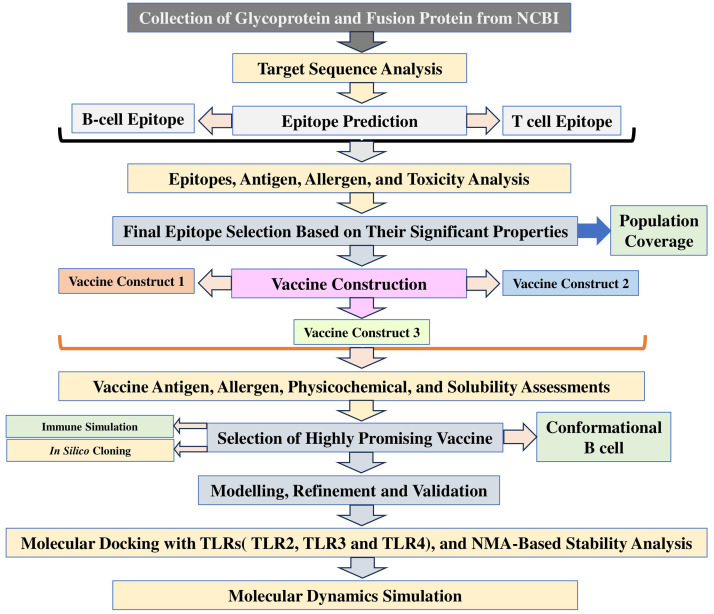
Illustration of the employed integrated immunoinformatic and computational-assisted steps to design the vaccine.

An immunoinformatics and bioinformatics-assisted step, integrated with computational methods, was employed in this study, and the steps were precisely followed to formulate a promising vaccine to combat NiV infection. The precise methodology and the steps employed are in Fig. 1.

### 2.1 Target sequence collection and its analysis

Given the vital role, two target sequences were obtained from NCBI (Sayers et al., 2025) for epitope prediction for vaccine construction. These target sequences were analysed using the VaxiJen v2.0 (Doytchinova and Flower, 2007) and ProtParam (Gasteiger et al., 2005) tools available in Expasy to assess their immunogenic and physicochemical features. In the VaxiJen v2.0-assisted assessment, viruses were considered as target organisms with a threshold of 0.4, whereas in ProtParam, the default parameters were used.

### 2.2 B and T cell epitope prediction and their assessments

In vaccine development, combining B- and T-cell responses targeting promising epitopes will help overcome viral infections. For the target's B-cell epitope, prediction was performed using Bepipred Linear Epitope Prediction 2.0, available in the IEDB (Jespersen et al., 2017), to identify a promising epitope that could trigger an immune response. Subsequently, these epitopes were examined via VaxiJen v2.0 (Doytchinova and Flower, 2007) and AllerTOP v.2.1 (Dimitrov et al., 2014) to identify suitable, antigenic, and non-allergenic promising epitopes. Moreover, the T cell epitope, followed by the MHC I and MHC II epitopes, was predicted via TepiTool (Paul et al., 2016), considering the most frequent restricted alleles to achieve high coverage. Furthermore, predicted epitopes were examined, like the antigen and allergen assessments described above. Additionally, ToxinPred (Gupta et al., 2013) was utilised to identify non-toxic epitopes within the predicted peptides.

### 2.3 Epitope selection, vaccine construction, and its assessments

A promising vaccine requires a vital, highly immunogenic epitope that can trigger an immune response to fight against the virus. Among the predicted epitopes, most are helpful because they possess the necessary properties, such as antigenicity, non-allergenicity, and non-toxicity. Regarding this, the promising epitope was selected from the predicted B and T-cell (MHCI and MHCII) epitopes for vaccine construction. Furthermore, a highly promising vaccine may yield significant outcomes by effectively triggering immune responses (Rahman et al., 2022; Sharma et al., 2025). Therefore, a total of 3 vaccines were constructed by combining different adjuvants. The MHC I was fused together with the AAY, and the MHC II was fused together with the GPGPG linker. Furthermore, the B cells were linked with the KK linkers, and the adjuvant was connected with the EAAAK linkers. Additionally, in the vaccine construct, His-Tag was linked via RVVR, and PADRE was utilised in vaccine construct. Moreover, across the different adjuvants, the remaining epitopes and linkers were kept constant, while only the adjuvants were varied in the construction of the three vaccine formulations. To assess the constructed vaccine's immune-associated properties, antigen, allergen, and physicochemical analyses were performed via VaxiJen v2.0 (Doytchinova and Flower, 2007), AllerTOP v.2.1 (Dimitrov et al., 2014), and ProtParam (Gasteiger et al., 2005). Furthermore, the solubility assessment was conducted via Protein–Sol (Hebditch et al., 2017).

### 2.4 Population coverage of vaccine candidates

To ensure that the higher population coverage may benefit from the formulated vaccine, the utilised epitopes have significant population coverage. The selected epitopes within both targets, along with their alleles, were provided as input, and the vaccine construct population was computed via Population Coverage (Bui et al., 2006) tool available in IEDB, using the combined approach with word coverage.

### 2.5 Structure modelling and validation

The constructed sequence was further used as input, and secondary structure were assesed via SOPMA (Geourjon and Deleage, 1995) and PSIPRED (McGuffin et al., 2000) servers to understand the contribution of structural elements of the sequence which is vital for analyzing their functions(Qian et al., 2025). Furthermore, the 3D structure of the constructed vaccine was modelled via Robetta (Baek et al., 2021). Subsequently, the model structure was enhanced using the GalaxyRefine (Heo et al., 2013) web server, and among the resulting models, the most promising were selected. Moreover, the model's stereochemical properties need to be favoured and significant; therefore, the final enhanced model was validated through PROCHECK (available in SAVES v6.1) (Laskowski et al., 1993), and the overall quality was assessed using ProSA-web (Wiederstein and Sippl, 2007) to understand the model structure of the constructed vaccine.

### 2.6 Conformation epitope prediction

The vaccine 3D model was further utilized to assess the location of B-cell conformational epitopes. The ElliPro (Ponomarenko et al., 2008) server was used to compute the epitope, considering the system-assisted default minimum score and distance, considering the vaccine model structure as input.

### 2.7 Docking with TLRs

Concerning the host immune activity, Toll-like receptors (TLRs) are vital; therefore, the vaccine model was further docked with the TLRs, such as TLR2, TLR3, and TLR4, through the ClusPro 2.0 server (Kozakov et al., 2017). The ClusPro 2.0 server is a rigid-body protein–protein docking platform that employs a blind (global) docking approach without prior specification of binding sites. Moreover, the server generated 30 different complex models based on the energy score. Among the complexes, the best-docked model was carefully chosen and used for binding affinity calculation via the PRODIGY (Xue et al., 2016) server; the free energy was also calculated via the HawkDock (Weng et al., 2019) server. Furthermore, the residue interaction activity was assessed via the PDBSum (Laskowski, 2001).

### 2.8 Simulation through NMA

Based on the docking analysis, the prospective TLRs with vaccine dowdmm complex stability were assessed through simulation, considering the iMODS (López-Blanco et al., 2014) tool, and also to examine the dynamic behavior of the complex. iMODS employed the normal mode analysis (NMA) to compute the collective motions of the complex.

### 2.9 Molecular dynamics simulation

The docked complexes from the docking analysis were subsequently used in simulations to assess their stability. The complex was considered as input, and the simulation was performed using the GROMACS software package, following the CHARMM36 force filed (Abraham et al., 2015; Du et al., 2026; Huang and MacKerell, 2013). The complex was placed in a cubic simulation box and solvated using the TIP3P water model under periodic boundary conditions, and appropriate counterions (Na⁺/Cl⁻) were added to neutralize the system.The energy minimization was performed following the steepest descent algorithm (Hatimuria et al., 2025; Sarkar et al., 2023). Further, the system underwent 2ns of equilibration, and the temperature and pressure were stabilized at 300K and 1 atm, considering the NVT and NPT ensembles (Iqbal et al., 2026; Nguyen and Kim, 2024). Finally, a 100 ns production run was carried out with a time step of 2 fs, and the trajectories were analyzed and visualized using RMSD, RMSF, and related metrics in VMD (Karimzadeh et al., 2025; Mishra et al., 2025).

### 2.10 Immune simulation and vaccine *in silico* assisted cloning

The formulated vaccine's immunological features were predicted using the C-IMMSIM (Rapin et al., 2011) web server to assess its ability to elicit immune activity and trigger an immune response. The vaccine model was used as an input, with a random seed of 12,345, a simulation volume of 50, and a total of 1050 simulation steps. Moreover, the formulated vaccines were simulated at different time intervals (1, 84, and 168) to simulate the 3 injections following the no lipopolysaccharide (LPS) condition, and the simulations were conducted accordingly (Kumar Mishra et al., 2025).

Furthermore, the vaccine sequence was used as input and optimised using the VectorBuilder Codon Optimization tool (https://en.vectorbuilder.com/) for *E. coli* (strain K12) to examine its expression level. The optimised vaccine sequence was imported into the pET-28a(+) plasmid using SnapGene software (https://www.snapgene.com/) and visualised accordingly.

## 3 Results

### 3.1 Target sequence collection and its analysis

In this investigation, two target proteins, i.e., Glycoprotein and the fusion protein, were examined as potential vaccine candidates. GenBank accession numbers QBQ56705.1 & QBQ56704.1, corresponding to the Glycoprotein and fusion protein, respectively, with sequence lengths of 602 and 546 amino acids, were obtained from NCBI. These sequences were analysed through VaxiJen v2.0 and ProtParam, and were found to have suitable properties. The complete feature activity and its analysis-based remarks are in **Supplementary Table 1**.

### 3.2 B and T cell epitope prediction and their assessments

The glycoprotein and fusion protein target sequences were used as input, and the linear epitope was predicted using BepiPred available in IEDB. The prediction identified 18 epitopes with lengths from 1 to 76 (**Supplementary Table 2**) and 22 epitopes with lengths from 1 to 23 (**Supplementary Table 3**) within the Glycoprotein and fusion protein, respectively. Additionally, epitopes of length 10 or more were considered among the predicted epitopes (6 for Glycoprotein and 11 for fusion protein) and analyzed. The immunogen-assisted analysis showed that, among the 6 epitopes, 4 are antigenic, 2 are non-antigenic, 4 are allergenic, and 2 are non-allergenic in the Glycoprotein (**Supplementary Table 4**), while among the 11 epitopes, 4 are antigenic, 7 are non-antigenic, 4 are allergenic, and 7 are non-allergenic in the fusion protein (**Supplementary Table 5**).

Furthermore, the T cell epitopes (MHC I and MHC II) were computed within the Glycoprotein and fusion protein target sequences using TepiTool, considering the most frequent alleles. The prediction revealed 162 MHC I and 65 MHC II epitopes in the Glycoprotein, and 169 MHCI and 60 MHC II epitopes in the fusion protein (**Supplementary Table 6-9**). Subsequently, the immunogen-assisted analysis showed that among the 162 MHCI epitopes, 85 are antigenic, 77 are non-antigenic, 81 are allergenic, and 81 are non-allergenic (**Supplementary Table 6**); among the 65 MHCII epitopes, 44 are antigenic, 21 are non-antigenic, 32 are allergenic, and 33 are non-allergenic within Glycoprotein (**Supplementary Table 7**), while 169 MHCI epitopes, 92 are antigenic, 77 are non-antigenic, 76 are allergenic, and 93 are non-allergenic (**Supplementary Table 8**); among the 60 MHCII epitopes, 30 are antigenic, 30 are non-antigenic, 30 are allergenic, and 30 are non-allergenic (**Supplementary Table 9**) within fusion protein computed via VaxiJen v2.0 and AllerTOP v.2.1. Moreover, the toxicity of the epitopes was computed via ToxinPred, and all identified epitopes were non-toxic.

### 3.3 Epitope selection and vaccine construction

Immunogen-assisted screening of predicted epitopes yielded several candidates; however, to refine the selection, the epitope with the highest antigenic score across both targets for B and T cells was chosen. Concerning the B cell epitope, a total 1 epitope within the Glycoprotein and 2 epitopes within the fusion protein were considered promising based on their properties as shown in Table 1. Furthermore, among the promising predicted MHC I and MHC II epitope, based on their immunogen-assisted properties within the both target protein, the Stringent criteria were incorporated, and the epitopes with at least 3 or more alleles along with the antigenic score 1 or higher were applied to select the epitopes having broad population coverage and highly antigenic, which may aid for a significant response (Kumar et al., 2021). Based on this, a total of 4 MHC I and 4 MHC II from Glycoprotein, and 9 MHC I and 3 MHC II from the fusion protein, were selected, as in Table 2.

**Table 1:** Selected B-cell epitope within the glycoprotein and fusion protein for vaccine construction.

**Position**	**Sequence**	**Antigenic and Score**
**Glycoprotein**		
6-43	KKVRFENTASDKGKNPSKVIKSYYGTMDIKKINEGLLD	0.6810 (Yes)
**Fusion Protein**		
323-332	ISNIEIGFCL	1.6889 (Yes)
523-543	NTYSRLEDRRVRPTSSGDLYY	0.7837 (Yes)

**Table 2:** Selected T cell (MHC I and II) epitope within the glycoprotein and fusion protein for vaccine construction.

**Position**	**Sequence**	**Percentile rank**	**Allele**	Antigen
**MHC I (Glycoprotein)**				
103-111	TEIGPKVSL	0.01	HLA-B*40:01 HLA-B*44:03 HLA-B*44:02	1.4043 (Yes)
118-126	ITIPANIGL	0.16	HLA-A*68:02 HLA-A*02:06 HLA-A*32:01 HLA-B*58:01 HLA-B*57:01	1.1090 (Yes)
199-207	KPKLISYTL	0.01	HLA-B*07:02 HLA-B*08:01 HLA-B*53:01 HLA-B*35:01 HLA-B*51:01	1.0819 (Yes)
452-460	VFYQASFSW	0.02	HLA-A*23:01 HLA-A*24:02 HLA-A*32:01 HLA-B*58:01 HLA-B*57:01 HLA-B*53:01	1.0092 (Yes)
**MHC II (Glycoprotein)**				
5-19	SKKVRFENTASDKGK	6.8	HLA-DRB1*03:01 HLA-DRB1*07:01 HLA-DRB1*15:01 HLA-DRB3*01:01 HLA-DRB3*02:02 HLA-DRB4*01:01 HLA-DRB5*01:01	1.0749 (Yes)
31-45	TMDIKKINEGLLDSK	12	HLA-DRB1*07:01 HLA-DRB1*15:01 HLA-DRB3*02:02 HLA-DRB4*01:01 HLA-DRB5*01:01	1.0167 (Yes)
455-469	QASFSWDTMIKFGDV	8	HLA-DRB1*03:01 HLA-DRB3*01:01 HLA-DRB3*02:02	1.1926 (Yes)
460-474	WDTMIKFGDVQTVNP	11	HLA-DRB1*07:01 HLA-DRB1*15:01 HLA-DRB3*01:01 HLA-DRB3*02:02 HLA-DRB4*01:01	1.0247 (Yes)
**MHC I (Fusion)**				
1-9	MAVILNKRY	0.12	HLA-B*35:01 HLA-B*53:01 HLA-A*26:01 HLA-B*58:01 HLA-A*30:02 HLA-B*57:01 HLA-B*15:01	1.5818 (Yes)
24-32	SVGILHYEK	0.19	HLA-A*11:01 HLA-A*03:01 HLA-A*30:01 HLA-A*68:01	1.6328 (Yes)
45-53	KYKIKSNPL	0.41	HLA-A*24:02 HLA-B*08:01 HLA-A*23:01 HLA-A*30:01	1.3144 (Yes)
114-122	IMAGVAIGI	0.28	HLA-A*02:03 HLA-A*02:01 HLA-A*32:01 HLA-A*02:06	1.0244 (Yes)
195-203	TELSLDLAL	0.05	HLA-B*40:01 HLA-B*44:03 HLA-B*44:02	1.1768 (Yes)
504-512	SLCIGLITF	0.22	HLA-A*32:01 HLA-B*15:01 HLA-A*23:01 HLA-A*24:02	1.4681 (Yes)
512-520	FISFIIVEK	0.12	HLA-A*68:01 HLA-A*11:01 HLA-A*03:01	1.1861 (Yes)
513-521	ISFIIVEKK	0.24	HLA-A*11:01 HLA-A*03:01 HLA-A*68:01 HLA-A*30:01	2.3237 (Yes)
517-525	IVEKKRNTY	0.15	HLA-A*30:02 HLA-A*01:01 HLA-B*15:01 HLA-A*32:01	1.5454 (Yes)
**MHC II (Fusion)**				
23-37	CSVGILHYEKLSKIG	15	HLA-DRB1*03:01 HLA-DRB1*15:01 HLA-DRB4*01:01	1.1148 (Yes)
117-131	GVAIGIATAAQITAG	17	HLA-DRB1*03:01 HLA-DRB1*07:01 HLA-DRB1*15:01 HLA-DRB3*01:01 HLA-DRB3*02:02 HLA-DRB4*01:01 HLA-DRB5*01:01	1.0404 (Yes)
513-527	ISFIIVEKKRNTYSR	4.5	HLA-DRB1*03:01 HLA-DRB3*02:02 HLA-DRB4*01:01 HLA-DRB5*01:01	1.2290 (Yes)

**Fig. 2:**
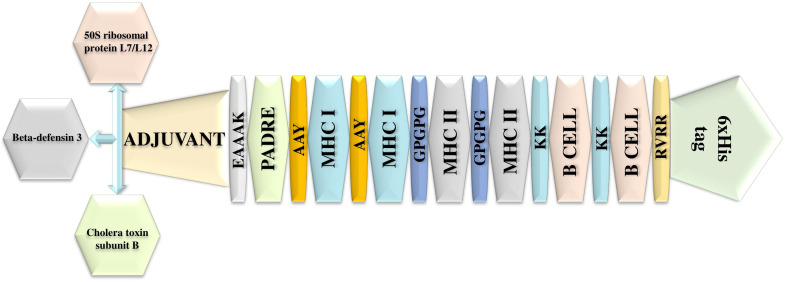
Illustration of epitopes yielding vaccine construction, along with other tributes.

Subsequently, these 3 B cells, 13 MHC I, and 7 MHC II (Combinely from both Glycoprotein and fusion) were used for the vaccine construction. Moreover, epitopes were analysed via Conservancy Analysis tool (Bui et al., 2007), and found that the epitopes were conserved, followed by their minimum and maximum identity. The 13 MHC I epitopes were attached with AAY linkers, whereas the 7 MHC II epitopes were attached through GPGPG linkers. Furthermore, the identified 3 B cell epitopes were linked with KK, whereas the 6 His-Tag was also attached at the C terminus and fused to the constructed sequence via RVRR linkers. Moreover, the 3 different adjuvants (50S ribosomal protein L7/L12, Beta-defensin 3, and CTB) were utilised for the 3 different vaccine formulations and were attached with the vaccine at the N-terminal individually to design 3 different vaccines (Vaccine Construct 1; 50S ribosomal protein L7/L12, Vaccine Construct 2; Beta-defensin 3, Vaccine Construct 3; CTB). Moreover, integrating these adjuvants into vaccine construction enhances the vaccine’s immunogenicity, promoting a stronger immune response, and they were reported as reliable in the reported study as well as in experimental evaluation (Bharucha et al., 2021; Kim et al., 2007). Additionally, the PADRE was also included in the vaccine to improve its effectiveness. The formulation of vaccine constructs is shown in Fig. 2. Moreover, based on the adjuvant variation in each construction, along with the remaining epitope and linker, the 3 formulated vaccines are shown in **Supplementary Fig.1.** These 3 vaccine-constructed sequences were further analysed via different servers (VaxiJen v 2.1, AllerTOP v.2.1, and ProParam), and the outcomes are shown in Table 3. The analysis revealed the significant outcome and properties of all the vaccines based on the utilised different adjuvants, which were antigenic and non-allergenic. Moreover, among these vaccines, the vaccine construct 2 (based on Beta-defensin 3) was further considered; it showed a higher antigenic score and a higher solubility score than the other two constructed vaccines. Moreover, β-defensin functions as a chemotactic agent and is promising for targeting sites of viral infection based on its mechanism (Ismail and Barakat, 2025).

**Table 3:** Three different vaccine features assessment and their properties.

**Feature**	**Designed Vaccine**		
	**Vaccine Construct 1**	**Vaccine Construct 2**	**Vaccine Construct 3**
**Antigencity (Score)**	Yes (0.7622)	**Yes (0.8554)**	Yes (0.8046)
**Allergen**	No	No	No
**Number of amino acids**	529	444	523
**Theoretical pI**	9.37	9.81	9.64
**Molecular weight**	56584.61	48305.32	57101.30
**Total number of negatively charged residues (Asp + Glu)**	53	30	39
**Total number of positively charged residues (Arg + Lys)**	72	69	70
**Formula**	C2581H4097N667O735S11	C2196H3483N593O602S15	C2609H4101N683O724S15
**Total number of atoms**	8091	6889	8132
**Estimated half-life**	30 hours (mammalian reticulocytes, in vitro).	30 hours (mammalian reticulocytes, in vitro).	30 hours (mammalian reticulocytes, in vitro).
	>20 hours (yeast, in vivo).	>20 hours (yeast, in vivo).	>20 hours (yeast, in vivo).
	>10 hours (Escherichia coli, in vivo)	>10 hours (Escherichia coli, in vivo).	>10 hours (Escherichia coli, in vivo)
**Instability index**	20.97	24.54	24.21
**Aliphatic index**	90.70	84.48	87.42
**Grand average of hydropathicity (GRAVY)**	-0.035	-0.166	-0.100
**Solubility**	0.483	0.513	0.423

### 3.4 Population coverage of vaccine candidates

**Fig. 3:**
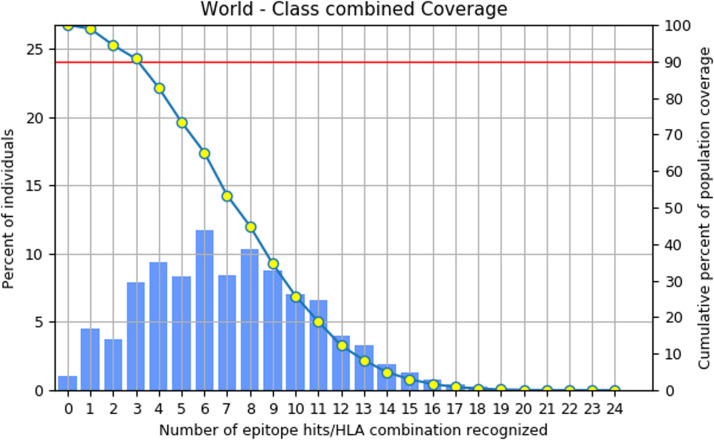
Representation of the word population chart based on the combined coverage.

Based on the employed steps, 13 MHC I and 7 MHC II were identified as promising and utilised in the vaccine formulation. Subsequently, these epitope-allele pairs were used as input, and the population coverage of the vaccine candidate was computed to accomplish high coverage of the utilised epitope for vaccine construction. The investigation revealed that the utilized epitopes have 98.97% coverage of the world population, based on the combined epitope, and are promising. Furthermore, the population chart is based on the utilized epitope region, as shown in Fig. 3.

### 3.5 Structure modelling and validation

**Fig. 4:**
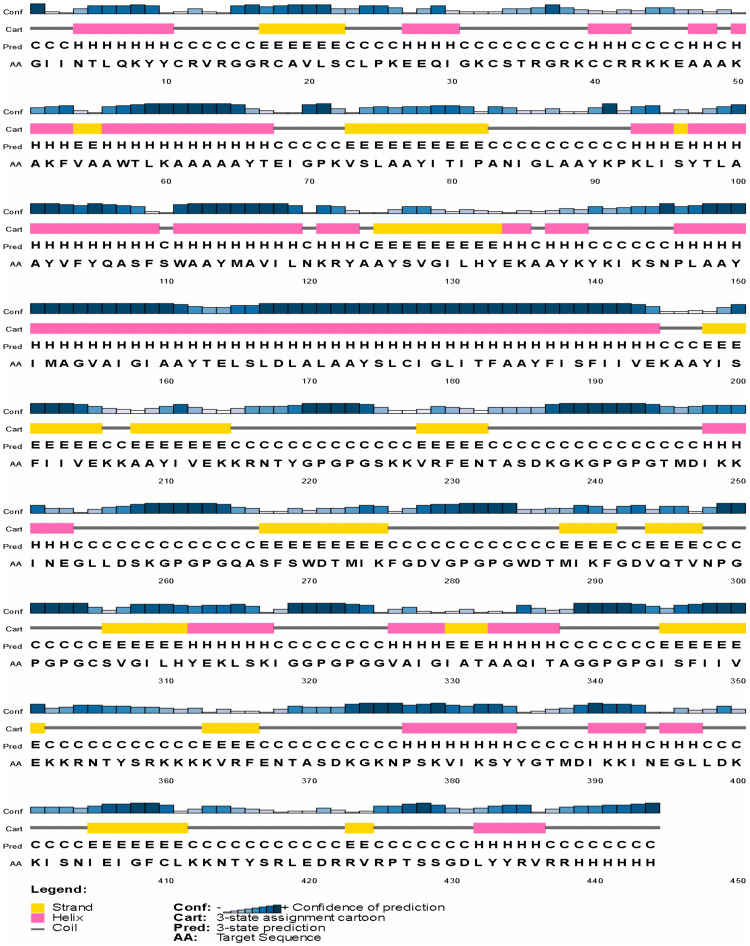
Illustration of the secondary structure of the vaccine constructed via the PSIPRED server.

The final selected constructed vaccine secondary structure was computed via the SOPMA and PSIPRED servers. The assessment revealed the vaccine construct has 20.05% helix, 26.13% strand, and 53.83% random coil. Moreover, PSIPRED was utilized to assess, as illustrated in Fig. 4, demonstrating various secondary features.

**Fig. 5:**
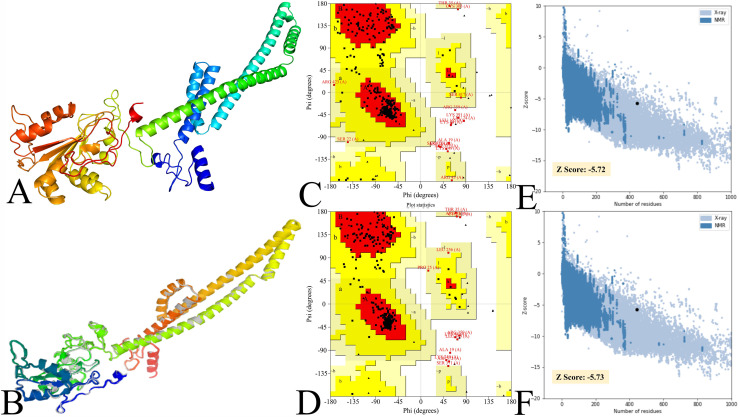
Illustration of the designed vaccine structure and its validation plot. (A and B) Initial and refined model structure (C and D ), Ramachandran plot of initial and refined model (E and F ), and Z-score plot of initial and refined model.

**Fig. 6:**
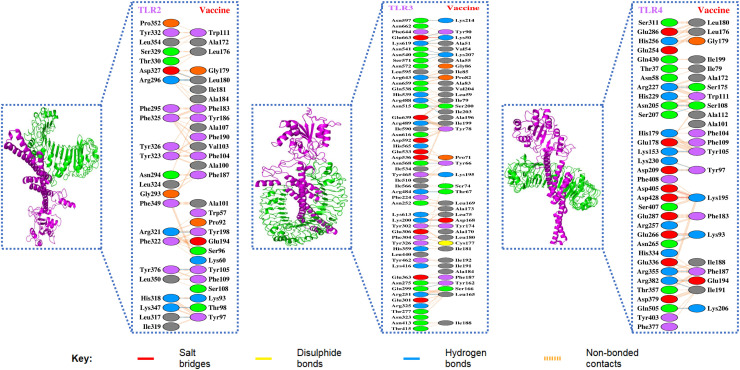
The interaction between TLRs (green color) and the vaccine (magenta) visualized using the PDBSum server.

Simultaneously, the vaccine construct 2 (VC2), comprising 444 amino acids, was subjected to 3D modeling using the Robetta server. The server generated 5 models for the queried vaccine sequence; however, all vaccines have a similar confidence score (i.e., 0.40), so Model 1 was selected. Model 1 was further enhanced using GlaxayRefine, and a 5-refined model was obtained based on the system-generated parameters to evaluate the enhanced model quality, as shown in Table 4. Moreover, the parameters, based on their scores, indicated that model 5 had superior quality and was selected for further analysis. A modelled structure should have significant quality, and concerning this, the initial (Fig. 5A) and final enhanced model (Fig. 5B) were validated via the PROCHECK and ProSA web. The PROCHECK-assisted Ramachandran plot of the vaccine revealed that the initial (Fig. 5C) and refined (Fig. 5D) vaccines have 85.95% and 89.1% favoured region, along with the promising Z score value (Fig. 5E: Initial model, Fig. 5F: Refined model), and further detail values and score are in Table 5. Moreover, concerning the diwallowed region-specific residues, which were initially 11 (2.9%), were reduced to 7 (1.9%) in the refined structure, and these are mainly located in flexible loops and terminal regions and are unlikely to affect structure quality significantly. Subsequently, the QMEAN4 server (Benkert et al., 2011) was also used to validate the structure, resulting in QMEAN4 scores of -3.18, indicating acceptable quality of the model (Mudipalli Elavarasu and K, 2025). Moreover, the Ramachandran and Z-score plots are shown in Fig. 6. The overall assessment demonstrated the reliability of the vaccine modelled structure.

**Table 4:** Enhanced vaccine model with generated value based on various parameters.

Model	GDT-HA	RMSD	MolProbity	Clash score	Poor rotamers	Rama favored
Initial	1.0000	0.000	1.658	3.7	0.0	91.6
MODEL 1	0.9769	0.330	1.953	10.7	0.0	93.9
MODEL 2	0.9702	0.355	1.926	10.2	0.0	94.1
MODEL 3	0.9769	0.325	1.989	10.8	0.0	93.2
MODEL 4	0.9724	0.361	2.033	11.8	0.3	93.0
**MODEL 5**	**0.9820**	**0.327**	**1.960**	**11.5**	**0.6**	**94.3**

**Table 5:** Plot statistics of the initial and refined vaccine modelled structure.

**Properties**	**Plot Static**	
	**Initial Vaccine Structure**	**Refined Vaccine Structure**
Residues in the most favoured regions	85.9%	89.1%
Residues in additional allowed regions	9.9%	8.0%
Residues in generously allowed regions	1.3%	1.1%
Residues in disallowed regions	2.9%	1.9%
Z Score	-5.72	-5.73

### 3.6 Conformational epitope prediction

The vaccine 3D structure was further subjected to ElliPro, and the conformational epitope within the vaccine was computed. The analysis revealed 7 epitopes, ranging in length from 3 to 71 residues, with scores from 0.55 to 0.802, as in Table 6. The presence of several conformational epitopes demonstrates that the vaccine has high quality and may yield significant outcomes.

**Table 6:** List of predicted epitopes within the formulated vaccine.

**No.**	**Residues**	**Number of residues**	**Score**
1	A:W111, A:A112, A:A113, A:Y114, A:M115, A:A116, A:V117, A:I118, A:L119, A:N120, A:K121, A:R122, A:Y123, A:A124, A:A125, A:Y126, A:S127, A:V128, A:G129, A:I130, A:L131, A:H132, A:Y133, A:E134, A:K135, A:A136, A:A137, A:Y138, A:K139, A:Y140, A:I142, A:K143, A:S144, A:N145, A:P146, A:L147, A:A148, A:A149, A:Y150, A:I151, A:M152, A:A153, A:G154, A:V155, A:A156, A:I157, A:G158, A:I159, A:A160, A:A161, A:Y162, A:T163, A:E164, A:L165, A:S166, A:L167, A:D168, A:L169, A:A170, A:L171, A:A172, A:A173, A:Y174, A:S175, A:L176, A:C177, A:I178, A:G179, A:L180, A:I181	70	0.802
2	A:G1, A:I2, A:I3, A:N4, A:T5, A:L6, A:Q7, A:K8, A:Y9, A:Y10, A:C11, A:R12, A:V13, A:R14, A:G15, A:G16, A:R17, A:C18, A:A19, A:V20, A:L21, A:S22, A:C23, A:L24, A:P25, A:K26, A:Q29, A:G31, A:K32, A:C33, A:S34, A:T35, A:G37, A:R38, A:K39, A:C40, A:C41, A:R42, A:R43	39	0.755
3	A:S269, A:W270, A:D271, A:I274, A:K275, A:G292, A:D293, A:Y312, A:E313, A:K314, A:S316, A:K317, A:I318, A:G319, A:G320, A:P321, A:G322, A:P323, A:E351, A:K352, A:K353, A:R354, A:N355, A:T356, A:Y357, A:S358, A:R359, A:K360, A:K361, A:K363, A:V364, A:R365, A:F366, A:E367, A:N368, A:T369, A:A370, A:S371, A:D372, A:K373, A:G374, A:K375, A:N376, A:P377, A:S378, A:K379, A:V380, A:I381, A:K382, A:S383, A:Y384, A:G386, A:T387, A:M388, A:D389, A:I390, A:K391, A:K392, A:I393, A:N394, A:E395, A:G396, A:L397, A:L398, A:D399, A:K400, A:I402, A:S403, A:N404, A:I405, A:I407	71	0.71
4	A:A331, A:R425, A:P426, A:T427, A:S428, A:S429, A:G430, A:D431, A:L432, A:Y433	10	0.608
5	A:D278, A:V279, A:G280, A:P281, A:G282, A:P283, A:W285, A:D286	8	0.598
6	A:G70, A:P71, A:K72	3	0.584
7	A:G342, A:P343, A:L411, A:K412, A:K413, A:N414, A:T415, A:R438, A:H440, A:H443, A:H444	11	0.55

### 3.7 Docking with TLRs

The final vaccine model was further docked with TLR2, TLR3, and TLR4 via the ClusPro server. Moreover, the structures of the TLRs were collected from PDB using the following IDs: TLR2 (6NIG), TLR3 (1ZIW), and TLR4 (4G8A). Toll-like receptors (TLRs) are vital in immune recognition and activation. In particular, TLR3 is involved in viral RNA sensing, while TLR2 and TLR4 recognize viral glycoproteins (Hanzelmann et al., 2016; Kayesh et al., 2023). Moreover, the vaccines do not typically act as direct ligands for TLRs; the docking analysis results underscore the need for further experimental evaluation to assess their effectiveness (Mishra et al., 2025d; Sharma et al., 2025). Their inclusion in this analysis provides computational insights into the binding pattern interactions between the vaccine and TLRs. The docking analysis revealed a total of 30 alternative docked complexes based on their score, as shown in **Supplementary Table 10** (Vaccine with TLR2), **Supplementary Table 11** (Vaccine with TLR3), and **Supplementary Table 12** (Vaccine with TLR4). Further, among the docked complexes, complexes having the lowest energy value among the models were selected and highlighted in **Supplementary Table 10-13**, which revealed that in the case of Vaccine-TLR2, Vaccine-TLR3, Vaccine-TLR4, complex 2, 0, and 18 were significant among others. The binding affinity of these complexes was computed via PRODIGY, which revealed that the model binding affinity ranged from -14.2 kcal/mol to -23.1 kcal/mol, and the free energy range from -121.25 kcal/mol to -165.94 kcal/mol. Moreover, the interactions and other properties of the complexes are shown in Table 7, whereas the complexes and the interaction residues are visualized in Fig. 6 (green represents the TLR and magenta represents the vaccine).

**Table 7:** Vaccine and TLR-based docking assessment and interaction properties.

**Properties**	**TLR2-Vaccine**	**TLR3-Vaccine**	**TLR4-Vaccine**
Energy Score	-1242.7	-1321.6	-1210.4
Binding Affinity (Kcal/mol)	-14.2	-23.1	-15.7
Free Energy (Kcal/mol)	-121.25	-165.94	-149.68
No. of interface residues	23res:28 res	52 res:41 res	33res:23 res
Interface area (Å2)	1602: 1526	2251:2440	1503:1575
No. of salt bridges		2	3
No. of hydrogen bonds	9	20	12
No. of non-bonded contacts	189	251	166

### 3.8 Simulation through NMA

**Fig. 7:**
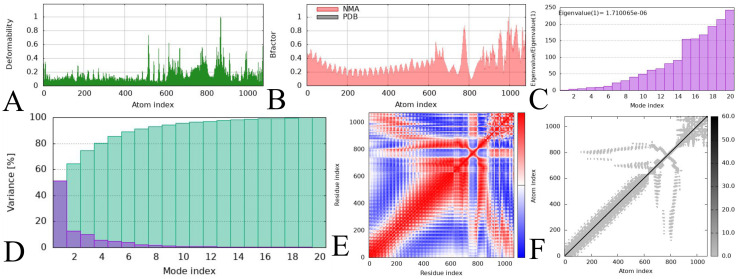
The NMA-assisted molecular simulation of TLR3 with the vaccine-docked complex. (A) Deformability, (B) B factor, (C) Eigenvalue, (D) Variance, (E) Covariance matrix, (F) Elastic Network.

Among the used TLR2, TLR3, and TLR4, the vaccine demonstrated significant activity against TLR3, as indicated by the number of H bonds. These docked TLR3-Vaccine complex stability analyses were performed on the iMODS server using the employed NMA to examine stability as a function of the complex's molecular modifications. The analysis demonstrates the deformability, which indicates the distortion of the residues (Fig. 7A), the examined B-factor based on the NMA and PDB (Fig. 7B), a total obtained eigenvalue ie., 1.710065e-06, which indicates easier deformation and represents the stiffness of the motion in the complex as in Fig. 7C, whereas Fig. 7D and 7E demonstrate the variance and covariance matrix, which determine the correlations, and lastly, Fig. 7F demonstrates the elastic network model, where the colour shown according to their stiffness.

### 3.9 Molecular dynamics simulation

**Fig. 8:**
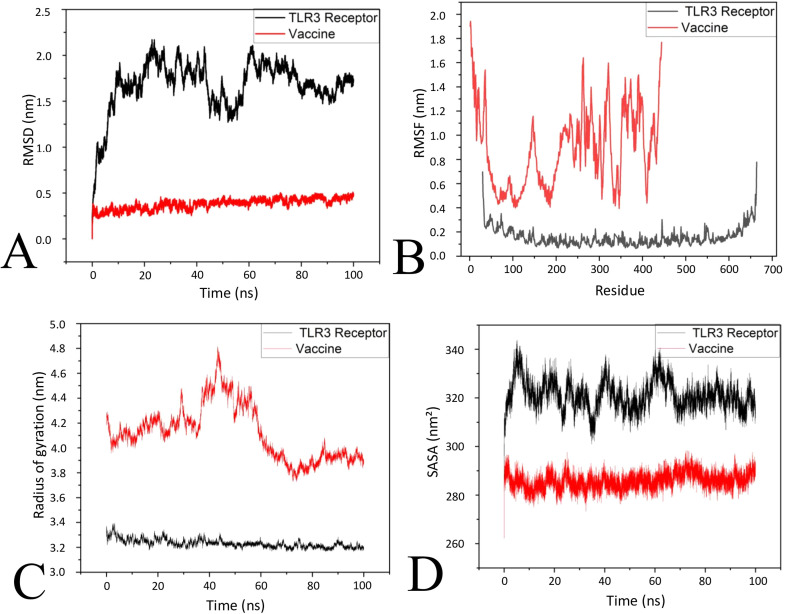
Simulation yielded stability analysis of the TLR3 and vaccine complex. (A) RMSD, (B) RMSF, (C) Rg and (D) SASA.

Based on the docking analysis, the TLR3-vaccine complex was found most promising based on the interactive residues, and this was further examined for stability analysis using GROMACS over 100 ns. The trajectories analysis revealed the promising compactness and stability of the docked complex. The RMSD analysis of the TLR3 and vaccine demonstrated the the stable conformational devaiation followed by the minimal conformational changes upon binding based on their value range from ∼1.4–2.1 nm for TLR3 and ∼0.25–0.48 nm as in Fig. 8A. The RMSF plot showed minor fluctuations across different regions following the range of ∼0.1–0.2 nm for the TLR3 and, ∼0.5– 1.9 nm for the vaccine. Based on the fluctuation of TLR3 suggesting the increases at terminal regions, whereas the a high flution were in the vaccine region suggesting the corresponding to the terminal regions and suggesting the stability of the complex as in Fig. 8B. Subsequently, most of the residues demonstrated low fluctuations and suggested the stability. The radius of gyration (Rg) plot (Fig. 8C) for the TLR3 value ranging from ∼3.2 to 3.4 nm which demonstrated the reliable structural compactness, whereas the for the vaccine the range was from ∼3.8 to 4.8 nm followed by the fluctuation in some region suggesting the conformational flexibility Lastly, the SASA analysis in Fig. 8D showed values fluctuating between ∼310 and 340 nm² for TLR3, whereas for the vaccine, the range was ∼280 to 300 nm², demonstrating consistent solvent exposure and minor fluctuations suggesting the stable interaction with the receptor.

Collectively, the docked complex exhibited overall stability, with minor fluctuations primarily localized to terminal and loop regions, reflecting normal conformational adjustments. Additionally, the non-covalent interactions analysis was accomplished through the COCOMAPS 2.0 server (Chawla et al., 2025) to examine the interactions, considering the first and final snapshots, which resulted in the promising interaction patterns as in **Supplementary Table 13**.

### 3.10 Immune simulation

**Fig. 9:**
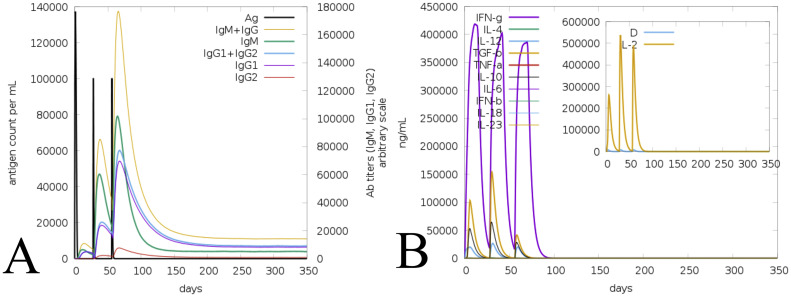
Vaccine-assisted immune response assessed via the C-Immsim server. (A) Immunoglobulins plots and their peaks on the time steps of injection, and (B) Cytokines and interleukins response.

A promising vaccine must be able to provoke an immune response to combat the infection and reduce its effects in individuals. Therefore, the vaccine-induced responses were computed via C-ImmSim server, accounting for the 3 injections at different time intervals. The assessed activity revealed the vaccine's ability to elicit the appropriate immune response, as shown in the Fig. 9. The immunoglobulin peaks represent high titers, followed by the concentrations of IgM and IgG reaching their highest levels, along with an immediate decline in the antigen count. Moreover, the vaccine interval showed increases in antibody levels, including IgM+IgG, IgM, IgG1+IgG2, etc as in Fig. 9A. Simultaneously, cytokines and interleukins also demonstrated a significant increase in levels as in Fig. 9B. The combined immunoglobulin, cytokine, and interleukin demonstrated substantial immune activity and the ability to induce an immune response.

### 3.11 *In silico*-assisted cloning and expression analysis

**Fig. 10:**
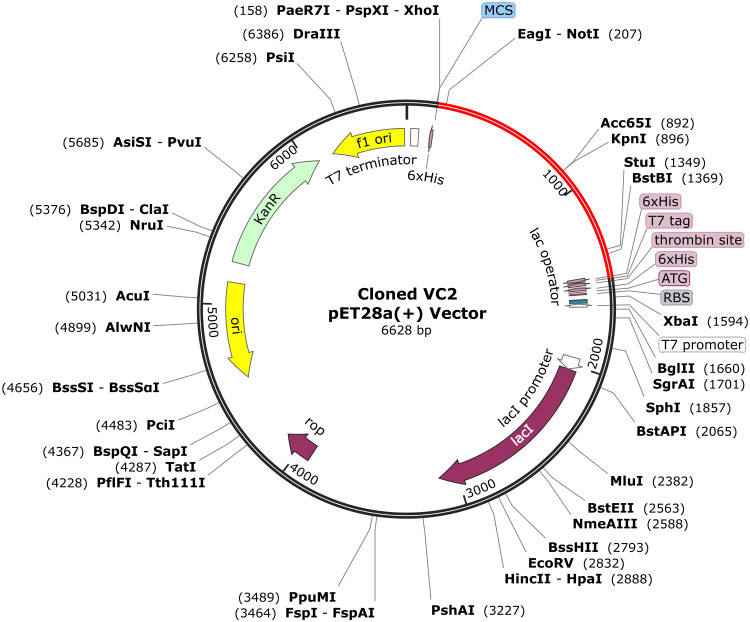
*In silico*-assisted vaccine cloning in the pET-28a(+) vector via SnapGene software. The red part indicates the incorporated vaccine.

The final VC2 comprised 444 aa, which was reverse-translated and optimised via the VectorBuilder Codon optimization tool, considering the *E. coli*. The vaccine optimization revealed a total sequence of 1335 nucleotides. Moreover, the CAI and GC% of the sequence were 0.94 and 51.16%, respectively, indicating a promising expression level, as the obtained values lie within the high expression range (Fahira et al., 2023; Sharma et al., 2025). Furthermore, the optimised sequence was incorporated into the pET-28a(+) vector using the SnapGene software, as shown in Fig. 10, considering the restriction site as previously reported, along with other steps (Banico et al., 2024; Basmenj et al., 2025).

## 4 Discussion

NiV is an ongoing health concern due to its severity and the frequent outbreaks, which lead to high transmission and fatality (Branda et al., 2025; Ganguly et al., 2025). To address this, no vaccine is available to completely prevent from this complication. However, over the past few years, vaccine development has been identified as the most promising strategy to tackle merging and re-emerging viral infections (De Groot et al., 2020; Excler et al., 2021; Wang et al., 2025; Weichel and Koch, 2024). Nevertheless, some adverse reactions caused by vaccines were also reported (Jia et al., 2023; Zhang et al., 2023). Moreover, compared to conventional strategies, at present, immunoinformatics, bioinformatics, reverse vaccinology, and advances *in silico*-assisted steps were found most suitable towards the successful vaccine design, and were keenly interested due to their time and cost effectiveness (Basmenj et al., 2025; Fadaka et al., 2021; Kaur et al., 2024; Shahab et al., 2024; Soltan et al., 2021).

Therefore, considering the glycoprotein and fusion protein as key target proteins, which are vital in host-pathogen interactions, in this study, an advanced integrated immunoinformatics and bioinformatics strategy was employed, along with the crucial steps of assessment and validation for the epitope-based vaccine development against the NiV virus. Multiple B and T cell (MHCI and MHC II) epitopes were predicted within the target; however, the epitopes were prioritized based on their high antigenic score along with restricted alleles, which will help achieve an immunodominant vaccine with high population cover, and a total of 13 MHC I, 7 MHC II, and 3 B cell epitopes were found suitable. The 23 epitopes were linked with different linkers, and their activity and features were enhanced with 3 different adjuvants, considering the PADRE and His-tag in all three vaccines. The activity of these three vaccines (VCA1, VC2, and VC3) was assessed, and VC2, which is designed based on the incorporated Beta-defensin 3 adjuvants, was found to be the most suitable based on the high antigenic score (i.e., 0.8554). Assessment of the formulated vaccine antigenicity is vital to understanding vaccine activity. Shabbir et al. reported the design vaccine have 0.6647 with adjuvants and 0.7624 without adjuvants (Shabbir et al., 2025), Kaur et al. reported the 0.7560 antigenic score of the designed vaccine (Kaur et al., 2024), Sharma et al. reported the 0.59, and 0.6071 antigenic score of NiV_1 and NiV_2 (Sharma et al., 2025), whereas Kumar et. reported the 0.5512 as antigenic score of the designed vaccine (Kumar et al., 2024). Overall, the obtained antigenic score highlights the significant effectiveness. Moreover, the design vaccine was non-allergenic and had the favoured physiological properties. Furthermore, the vaccine solubility was 0.513, which is higher and similar to the previously reported studies (Kumar et al., 2024; Oladipo et al., 2025), demonstrating a suitable solubility score and higher solubility than 0.45 (Hebditch et al., 2017). The prioritised epitope vaccine must demonstrate high population coverage to support broader vaccine coverage. The formulated vaccine comprised 13 MHC I and 7 MHC II alleles, which together provided 98.97% word population coverage. The assessment of vaccine population coverage is vital, and the obtained coverage is nearly similar and outperforms the prior reported studies (Kaur et al., 2024; Rahman et al., 2022; Shabbir et al., 2025). The 2D assessment of the designed vaccine demonstrated a predominantly flexible, which may enhance epitope accessibility. Furthermore the enhanced model of the formulated vaccine revealed 89.1% as the most favorable region, which was nearly similar and higher to the 86.0 % (Shahab et al., 2024), 86.4% (Kaur et al., 2024), and 91.3% of NiV_1 and 90.5% of NiV_2 (Sharma et al., 2025), and 84.4 % (Masum et al., 2024) ensured remarkable model quality and reliability. The overall quality (Z-score) of the initial and refined model was -5.72 to -5.73, which were nearly similar and higher than the earlier reported study (Banico et al., 2024; Shahab et al., 2024; Sharma et al., 2025), whereas the low negative score indicates the structure reliability (Wiederstein and Sippl, 2007). The identified 7 conformational B cell epitope, comprising 212 aa, indicates the vaccine's strong immunodominant feature, and the presence of these B cells within the vaccine suggests its possible ability to elicit antibody binding and potentially activate the immune response. Moreover, the conformational B cell is vital for vaccine development, as it reflects the native structure, which is crucial for inducing neutralizing antibodies (Basmenj et al., 2025; Bulla et al., 2025). Targeting Toll-Like Receptors (TLRs) in vaccine development is vital to provide a signal to induce an effective immune response (Bzowka et al., 2023; Malik et al., 2023). The formulated vaccines were docked with TLRs, including TLR2, TLR3, and TLR4, to assess their binding patterns. Moreover, targeting these TLRs in vaccine development was found promising due to their crucial role and ability to recognize foreign substances (Rahman et al., 2022; Shahab et al., 2024; Sharma et al., 2025). The docking investigation demonstrates that the vaccine binds more interactively to TLR3 than to TLR2 or TLR4, based on its binding affinity (-23.1 kcal/mol), followed by 20 H-bonds. Moreover, regarding TLR3, Sharma et al. reported that the formulated NiV_1 and NiV_2 vaccines formed 32 and 10 hydrogen bonds, respectively (Sharma et al., 2025), whereas Masum et al. reported that the designed vaccine formed 11 and 15 hydrogen bonds with TLR2 and TLR4, respectively (Masum et al., 2024). The docking yielded an interaction study showing that the vaccine binds to TLRs; however, experimental evaluation is required to translate this interaction into functional immune effects. The Vaccine with TLR3 docked complex stability was assessed via the NMA-assisted approach, which demonstrates the stable molecular motion. The obtained eigenvalue of 1.710065e-06 indicates the low deformability. Subsequently, Shabbir et al. reported the eigenvalue of 1.066802e-08 (Shabbir et al., 2025), whereas Soltan et al. reported 1.63 × 10−5 as the eigenvalue (Soltan et al., 2021). Along with the other simulated parameters, the complex stability details of the obtained eigenvalue demonstrated the reliability of the vaccine and the TLR-docked complex. Moreover, the simulation over 100ns, followed by the RMSD, RMSF, RG, and SASA, resulted in remarkable stability. Furthermore, the formulated vaccine was simulated at different time intervals (1, 84, and 168) and found to reduce antigen levels and induce strong immune activation. The high levels of immunoglobulins and the demonstrated exposure indicated strong antibody responses and significant immune activation, followed by strong immunological memory. At the same time, the multiple cytokine levels demonstrated the significant cellular immune activity. The combined generation of immunoglobulins, cytokines, and interleukins suggests the possible ability to induce an effective immune response (Oladipo et al., 2025; Shabbir et al., 2025). Moreover, the *in silico*-assisted cloning and their expression in the pET28a (+) vector were found to be a suitable step towards the expression analysis of vaccine. The design vaccine revealed the CAI value 0.94 and GC% 51.16, considering the *E. coli*. Moreover, the CAI value and GC from 0.8–1.0 and 30%–70% indicate the optimal expression (Chai et al., 2025). Regarding the expression analysis, Sharma et al. reported 53.85% (Sharma et al., 2025), 52.2% (Das et al., 2024), and 45.36% (Masum et al., 2024), and the obtained values were nearly aligned and suggest adequate expression levels of the formulated vaccine. Overall, the integrated steps yielded a vaccine that revealed significant immunodominant features and demonstrated its ability to induce an effective immune response.

### 4.1 Limitations of the study

The formulated vaccine demonstrated remarkable activity; however, the study has certain limitations. The integrated advanced immunoinformatics and bioinformatics approach identified immunodominant B- and T-cell epitopes, which were incorporated into the vaccine, and the final constructed vaccine, which relied on multiple steps involving tools, servers, and databases, remains promising. The results remain predictive, based on the implementation of computational resources. Therefore, comprehensive experimental evaluation followed by *in vivo* and *in vitro* activity is required to ensure the formulated vaccine's activity, the induced immune activity, and its ability to combat viral infection.

## 5 Conclusion

Concerning the ongoing NiV infection, this investigation employed an integrated immunoinformatics and bioinformatics approach to formulate a highly antigenic multi-epitope vaccine (evaluated with different adjuvants to enhance vaccine activity) with immunodominant activity, thereby protecting against NiV. The formulated vaccine will elicit both humoral and cellular immunity through a combination of highly immunodominant B- and T-cell epitopes, along with a selected adjuvant. The docking analysis of the formulated vaccine to TLR2, TLR3, and TLR4 demonstrated promising interaction, with binding affinities ranging from -14.2 kcal/mol to -15.7 kcal/mol. Among these, the vaccine–TLR3 complex showed comparatively stable interactions through hydrogen-bonding patterns; however, these findings should be interpreted as exploratory rather than indicative of direct receptor targeting. NMA and simulation analysis resulted the stability of the TLR and vaccine. Furthermore, the *in silico* assisted vaccine-induced activity demonstrated the ability to generate strong immune responses, followed by a reduction in antigen levels over time after injection. Overall, the integrated computational framework provides a rational strategy for multi-epitope vaccine design against NiV and may serve as a useful approach for other diseases. Collectively, the outcomes of this study are based on computational steps and require *in vitro* and *in vivo* experimental validation to ensure the effectiveness and findings.

## Declaration of generative AI and AI-assisted technologies in the manuscript preparation process

During the preparation of this work, the author(s) used ChatGPT for only language editing and grammar correction to improve readability. After using this tool/service, the author(s) reviewed and edited the content as needed and take(s) full responsibility for the content of the published article.

## CRediT authorship contribution statement

**Waheeb Sami Aggad:** Conceptualization, Writing – original draft, Visualization, Software, Methodology, Investigation, Formal analysis, Data curation. **Saurav Kumar Mishra:** Writing – original draft, Methodology, Investigation, Data curation. **Taha Alqahtani:** Writing – original draft, Project administration, Methodology, Investigation. **Abdelkrim Guendouzi:** Software, Resources, Methodology, Investigation. **Zsolt Tóth:** Writing – review & editing, Supervision, Resources, Project administration. **Magdi E.A. Zaki:** Writing – review & editing, Visualization, Supervision, Resources, Project administration, Methodology.

## Declaration of competing interest

The authors declare that they have no known competing financial interests or personal relationships that could have appeared to influence the work reported in this paper.

## Data Availability

All generated and analysed data were included in the manuscript/ supplementary file.

## Appendix Supplementary materials

Image, application 1
