# Supplementary material for: Epitope-based vaccine of NiV targeting glycoprotein and fusion protein: an integrated immunoinformatics and bioinformatics approach
Source: Virus Res. 2026 May 15;369:199746. doi: 10.1016/j.virusres.2026.199746 (PMC13218253; doi:10.1016/j.virusres.2026.199746)
Supplement: Supplementary file 1 [file mmc1.docx]

**Supplementary Table 1.** List of different assessed features of the target protein.

| **Feature** | **Target Protein Name** | |
| --- | --- | --- |
|  | **Glycoprotein** | **Fusion** |
| **Antigencity (Score)** | 0.5100 (Probable ANTIGEN) | 0.4893 (Probable ANTIGEN) |
| **Number of amino acids** | 602 aa | 546 aa |
| **Theoretical pI** | 8.16 | 6.08 |
| **Molecular weight** | 67179.06 | 60280.90 |
| **Total number of negatively charged residues (Asp + Glu)** | 56 | 48 |
| **Total number of positively charged residues (Arg + Lys)** | 60 | 46 |
| **Formula** | C2997H4730N788O901S30 | C2687H4361N693O817S26 |
| **Total number of atoms** | 9446 | 8584 |
| **Estimated half-life** | 30 hours (mammalian reticulocytes, in vitro). >20 hours (yeast, in vivo). >10 hours (Escherichia coli, in vivo). | 30 hours (mammalian reticulocytes, in vitro). >20 hours (yeast, in vivo). >10 hours (Escherichia coli, in vivo). |
| **Instability index** | 35.91 | 38.05 |
| **Aliphatic index** | 89.00 | 112.27 |
| **Grand average of hydropathicity(GRAVY)** | -0.194 | 0.177 |

**Supplementary Table 2.** Predicted epitope within the glycoprotein, along with sequence and length.

| **No.** | **Start** | **End** | **Epitope Sequence** | **Length** |
| --- | --- | --- | --- | --- |
| 1 | 6 | 43 | KKVRFENTASDKGKNPSKVIKSYYGTMDIKKINEGLLD | 38 |
| 2 | 75 | 81 | RSTDNQA | 7 |
| 3 | 91 | 92 | QQ | 2 |
| 4 | 95 | 99 | KGLAD | 5 |
| 5 | 102 | 102 | G | 1 |
| 6 | 110 | 110 | S | 1 |
| 7 | 139 | 214 | NENVNEKCKFTLPPLKIHECNISCPNPLPFREYKPQTEGVSNLVGLPNNICLQKTSNQILKPKLISYTLPVVGQSG | 76 |
| 8 | 239 | 245 | SCSRGVS | 7 |
| 9 | 259 | 262 | GDEV | 4 |
| 10 | 273 | 277 | PSNPN | 5 |
| 11 | 300 | 313 | VGDPILNSTYWSGS | 14 |
| 12 | 325 | 342 | NNGESYNQHQFALRNIEK | 18 |
| 13 | 371 | 405 | VRTEFKYNDSNCPVAKCQYSKPENCRLSMGIRPNS | 35 |
| 14 | 420 | 423 | DEEN | 4 |
| 15 | 461 | 461 | D | 1 |
| 16 | 482 | 498 | NTVISRPGQSQCPRFNT | 17 |
| 17 | 530 | 532 | QTA | 3 |
| 18 | 554 | 558 | EDTNA | 5 |

**Supplementary Table 3.** Predicted epitope within the fusion protein, along with sequence and length.

| **No.** | **Start** | **End** | **Epitope Sequence** | **Length** |
| --- | --- | --- | --- | --- |
| 1 | 25 | 33 | VGILHYEKL | 9 |
| 2 | 43 | 44 | TR | 2 |
| 3 | 49 | 52 | KSNP | 4 |
| 4 | 65 | 74 | VSNMSQCTGS | 10 |
| 5 | 76 | 76 | M | 1 |
| 6 | 98 | 107 | KNNTHDLVGD | 10 |
| 7 | 142 | 142 | D | 1 |
| 8 | 156 | 156 | E | 1 |
| 9 | 188 | 195 | DKISCKQT | 8 |
| 10 | 215 | 226 | GPNLQDPVSNSM | 12 |
| 11 | 236 | 238 | GGN | 3 |
| 12 | 240 | 262 | ETLLRTLGYATEDFDDLLESDSI | 23 |
| 13 | 323 | 332 | ISNIEIGFCL | 10 |
| 14 | 342 | 351 | QDYATPMTNN | 10 |
| 15 | 353 | 354 | RE | 2 |
| 16 | 357 | 357 | T | 1 |
| 17 | 360 | 375 | TEKCPRELVVSSHVPR | 16 |
| 18 | 403 | 403 | Q | 1 |
| 19 | 433 | 446 | LGSVNYNSEGIAIG | 14 |
| 20 | 448 | 457 | PVFTDKVDIS | 10 |
| 21 | 468 | 484 | QQSKDYIKEAQRLLDTV | 17 |
| 22 | 523 | 543 | NTYSRLEDRRVRPTSSGDLYY | 21 |

**Supplementary Table 4.** Selected epitope within the glycoprotein, along with length, antigen, and allergen properties.

| **Position** | **Epitope Sequence** | **Length** | **Antigen** | **Allergen** |
| --- | --- | --- | --- | --- |
| **6-43** | **KKVRFENTASDKGKNPSKVIKSYYGTMDIKKINEGLLD** | **38** | **0.6810 (Yes)** | **No** |
| 139-214 | NENVNEKCKFTLPPLKIHECNISCPNPLPFREYKPQTEGVSNLVGLPNNICLQKTSNQILKPKLISYTLPVVGQSG | 76 | 0.5413 (Yes) | Yes |
| 300-313 | VGDPILNSTYWSGS | 14 | 0.0942 (No) | No |
| 325-342 | NNGESYNQHQFALRNIEK | 18 | 0.4381 (Yes) | Yes |
| 371-405 | VRTEFKYNDSNCPVAKCQYSKPENCRLSMGIRPNS | 35 | 0.4515 (Yes) | Yes |
| 482-498 | NTVISRPGQSQCPRFNT | 17 | -0.2478 (No) | Yes |

(Probable ANTIGEN: Yes; Probable NON-ANTIGEN: No; Probable ALLERGEN: Yes, Probable NON-ALLERGEN: No  )

**Supplementary Table 5.** Predicted epitope within the fusion protein, along with length, antigen, and allergen properties.

| **Position** | **Peptide** | **Length** | **Antigen** | **Allergen** |
| --- | --- | --- | --- | --- |
| 65-74 | VSNMSQCTGS | 10 | -0.2228 (No) | No |
| 98-107 | KNNTHDLVGD | 10 | 0.2964 (No) | Yes |
| 215-226 | GPNLQDPVSNSM | 12 | 0.1771 (No) | No |
| 240-262 | ETLLRTLGYATEDFDDLLESDSI | 23 | 0.0829 (No) | Yes |
| **323-332** | **ISNIEIGFCL** | **10** | **1.6889 (Yes)** | **No** |
| 342-351 | QDYATPMTNN | 10 | 0.3195 (No) | No |
| 360-375 | TEKCPRELVVSSHVPR | 16 | -0.1785 (No) | No |
| 433-446 | LGSVNYNSEGIAIG | 14 | 1.3971 (Yes) | Yes |
| 448-457 | PVFTDKVDIS | 10 | 0.7450 (Yes) | Yes |
| 468-484 | QQSKDYIKEAQRLLDTV | 17 | 0.1631 (No) | No |
| **523-543** | **NTYSRLEDRRVRPTSSGDLYY** | **21** | **0.7837 (Yes)** | **No** |

(Probable ANTIGEN: Yes; Probable NON-ANTIGEN: No; Probable ALLERGEN: Yes, Probable NON-ALLERGEN: No  )

**Supplementary Table 6.** Identified MHC I epitope within the Glycoprotein, along with other properties.

| **Position** | **Peptide** | **Percentile rank** | **Allele** | **Antigen** | **Allergen** | **Toxic** |
| --- | --- | --- | --- | --- | --- | --- |
| **7-15** | **KVRFENTAS** | **0.68** | **HLA-A*30:01** | **1.3030 (Yes)** | **(No)** | **No** |
| 17-25 | KGKNPSKVI | 0.83 | HLA-A*30:01 | 0.9122 (Yes) | (Yes) | No |
| 20-28 | NPSKVIKSY | 0.01 | HLA-B*35:01  HLA-B*53:01  HLA-A*26:01  HLA-B*07:02  HLA-B*44:02  HLA-B*44:03 | -0.3061 (No) | (No) | No |
| 21-29 | PSKVIKSYY | 0.62 | HLA-A*30:02  HLA-A*01:01 | -0.2269 (No) | (No) | No |
| 24-32 | VIKSYYGTM | 0.87 | HLA-B*08:01 | -0.1549 (No) | (No) | No |
| 26-34 | KSYYGTMDI | 0.43 | HLA-A*32:01 | 0.5366 (Yes) | (Yes) | No |
| 27-35 | SYYGTMDIK | 0.83 | HLA-A*30:01 | 1.8345 (Yes) | (Yes) | No |
| 33-41 | DIKKINEGL | 0.28 | HLA-A*68:02  HLA-B*08:01  HLA-A*26:01 | 0.6332 (Yes) | (Yes) | No |
| 38-46 | NEGLLDSKI | 0.7 | HLA-B*44:02  HLA-B*44:03  HLA-B*40:01 | 0.8836 (Yes) | (Yes) | No |
| 41-49 | LLDSKILSA | 0.08 | HLA-A*02:01  HLA-A*02:03  HLA-A*02:06  HLA-B*08:01  HLA-A*01:01 | 0.4034 (Yes) | (Yes) | No |
| 45-53 | KILSAFNTV | 0.06 | HLA-A*02:06  HLA-A*32:01  HLA-A*02:01  HLA-A*02:03  HLA-A*30:01 | 0.0273 (No) | (No) | No |
| 46-54 | ILSAFNTVI | 0.42 | HLA-A*02:03  HLA-A*02:01  HLA-A*32:01 | -0.1420 (No) | (No) | No |
| 48-56 | SAFNTVIAL | 0.19 | HLA-B*51:01  HLA-B*35:01  HLA-A*68:02  HLA-A*02:06  HLA-A*32:01  HLA-B*08:01  HLA-A*26:01  HLA-B*53:01  HLA-B*07:02 | 0.3171 (No) | (No) | No |
| 49-57 | AFNTVIALL | 0.35 | HLA-A*23:01  HLA-A*24:02 | 0.2781 (No) | (No) | No |
| 52-60 | TVIALLGSI | 0.29 | HLA-A*68:02  HLA-A*26:01 | 0.3438 (No) | (No) | No |
| **54-62** | **IALLGSIVI** | **0.16** | **HLA-B*51:01** | **0.5470 (Yes)** | **(No)** | **No** |
| 55-63 | ALLGSIVII | 0.14 | HLA-A*02:01  HLA-A*02:06  HLA-A*02:03  HLA-A*32:01 | 0.3957 (No) | (No) | No |
| 56-64 | LLGSIVIIV | 0.32 | HLA-A*02:01  HLA-A*02:03 | 0.2388 (No) | (Yes) | No |
| **59-67** | **SIVIIVMNI** | **0.94** | **HLA-A*68:02** | **0.7998 (Yes)** | **(No)** | **No** |
| 65-73 | MNIMIIQNY | 0.39 | HLA-A*26:01  HLA-A*30:02  HLA-B*35:01 | 0.4923 (Yes) | (Yes) | No |
| **67-75** | **IMIIQNYTR** | **0.28** | **HLA-A*31:01**  **HLA-A*33:01** | **0.4493 (Yes)** | **(No)** | **No** |
| 75-83 | RSTDNQAMI | 0.82 | HLA-B*58:01 | 0.3088 (No) | (Yes) | No |
| 76-84 | STDNQAMIK | 0.08 | HLA-A*11:01  HLA-A*01:01  HLA-A*03:01 | 0.0091 (No) | (Yes) | No |
| 79-87 | NQAMIKDAL | 0.86 | HLA-B*40:01 | -0.2686 (No) | (No) | No |
| 82-90 | MIKDALQSI | 0.1 | HLA-A*02:03  HLA-A*02:06  HLA-A*68:02  HLA-B*08:01  HLA-A*32:01  HLA-A*26:01  HLA-B*51:01  HLA-A*30:01  HLA-A*02:01 | -0.0616 (No) | (Yes) | No |
| 86-94 | ALQSIQQQI | 0.07 | HLA-A*02:03  HLA-A*02:01  HLA-A*32:01  HLA-A*02:06 | 0.2113 (No) | (No) | No |
| 92-100 | QQIKGLADK | 0.88 | HLA-A*11:01 | -0.2548 (No) | (Yes) | No |
| 97-105 | LADKIGTEI | 0.33 | HLA-B*51:01  HLA-B*53:01 | 0.1990 (No) | (Yes) | No |
| 100-108 | KIGTEIGPK | 0.26 | HLA-A*03:01  HLA-A*11:01 | 1.3100 (Yes) | (Yes) | No |
| 101-109 | IGTEIGPKV | 0.44 | HLA-B*51:01 | 1.2287 (Yes) | (Yes) | No |
| **103-111** | **TEIGPKVSL** | **0.01** | **HLA-B*40:01**  **HLA-B*44:03**  **HLA-B*44:02** | **1.4043 (Yes)** | **(No)** | **No** |
| 104-112 | EIGPKVSLI | 0.24 | HLA-A*68:02  HLA-B*08:01  HLA-A*26:01 | 1.3744 (Yes) | (Yes) | No |
| **110-118** | **SLIDTSSTI** | **0.04** | **HLA-A*02:03**  **HLA-A*02:01**  **HLA-A*02:06**  **HLA-A*32:01**  **HLA-B*15:01** | **0.6210 (Yes)** | **(No)** | **No** |
| 114-122 | TSSTITIPA | 0.48 | HLA-A*68:02 | 0.1786 (No) | (Yes) | No |
| 116-124 | STITIPANI | 0.04 | HLA-A*68:02  HLA-A*32:01  HLA-A*02:06  HLA-A*26:01  HLA-B*58:01 | 0.0143 (No) | (Yes) | No |
| **118-126** | **ITIPANIGL** | **0.16** | **HLA-A*68:02**  **HLA-A*02:06**  **HLA-A*32:01**  **HLA-B*58:01**  **HLA-B*57:01** | **1.1090 (Yes)** | **(No)** | **No** |
| **119-127** | **TIPANIGLL** | **0.9** | **HLA-A*26:01** | **1.1538 (Yes)** | **(No)** | **No** |
| 128-136 | GSKISQSTA | 0.47 | HLA-A*30:01 | 0.1740 (No) | (No) | No |
| 130-138 | KISQSTASI | 0.16 | HLA-A*32:01  HLA-A*02:03 | 0.1792 (No) | (Yes) | No |
| 134-12 | STASINENV | 0.01 | HLA-A*68:02  HLA-A*02:06  HLA-A*02:03 | 0.1186 (No) | (No) | No |
| 137-145 | SINENVNEK | 0.05 | HLA-A*11:01  HLA-A*03:01  HLA-A*30:01  HLA-A*68:01 | 0.0030 (No) | (No) | No |
| 147-155 | KFTLPPLKI | 0.19 | HLA-A*24:02  HLA-A*23:01 | 1.2406 (Yes) | (Yes) | No |
| 150-158 | LPPLKIHEC | 0.3 | HLA-B*08:01  HLA-B*51:01 | -0.1374 (No) | (No) | No |
| 160-168 | ISCPNPLPF | 0.13 | HLA-B*58:01  HLA-B*57:01  HLA-A*32:01  HLA-A*23:01  HLA-B*35:01  HLA-B*15:01  HLA-A*24:02  HLA-B*53:01 | 0.9723 (Yes) | (Yes) | No |
| 161-169 | SCPNPLPFR | 0.91 | HLA-A*31:01 | 0.8511 (Yes) | (Yes) | No |
| 169-177 | REYKPQTEG | 0.73 | HLA-B*40:01 | 0.6198 (Yes) | (Yes) | No |
| **170-178** | **EYKPQTEGV** | **0.9** | **HLA-A*24:02** | **1.2258 (Yes)** | **(No)** | **No** |
| 174-182 | QTEGVSNLV | 0.2 | HLA-A*68:02  HLA-A*01:01 | 0.1171 (No) | (No) | No |
| 176-184 | EGVSNLVGL | 0.63 | HLA-A*68:02 | 0.0634 (No) | (Yes) | No |
| 180-188 | NLVGLPNNI | 0.49 | HLA-A*02:03  HLA-A*02:01 | 0.1587 (No) | (Yes) | No |
| 190-198 | LQKTSNQIL | 0.17 | HLA-B*15:01 | 0.2476 (No) | (No) | No |
| 193-201 | TSNQILKPK | 0.09 | HLA-A*11:01  HLA-A*30:01  HLA-A*03:01  HLA-A*68:01 | -0.0368 (No) | (Yes) | No |
| 195-203 | NQILKPKLI | 0.55 | HLA-B*08:01 | -0.3690 (No) | (Yes) | No |
| 197-205 | ILKPKLISY | 0.01 | HLA-B*15:01  HLA-A*30:02  HLA-A*32:01  HLA-B*08:01  HLA-A*03:01  HLA-A*30:01  HLA-A*26:01  HLA-A*01:01  HLA-B*35:01  HLA-B*57:01 | 0.8331 (Yes) | (Yes) | No |
| **199-207** | **KPKLISYTL** | **0.01** | **HLA-B*07:02**  **HLA-B*08:01**  **HLA-B*53:01**  **HLA-B*35:01**  **HLA-B*51:01** | **1.0819 (Yes)** | **(No)** | **No** |
| 201-209 | KLISYTLPV | 0.05 | HLA-A*02:03  HLA-A*02:01  HLA-A*02:06  HLA-A*32:01 | 0.1202 (No) | (Yes) | No |
| **202-210** | **LISYTLPVV** | **0.76** | **HLA-A*02:06**  **HLA-A*02:03**  **HLA-A*02:01** | **0.5110 (Yes)** | **(No)** | **No** |
| 216-224 | CITDPLLAM | 0.44 | HLA-A*26:01 | 0.2359 (No) | (No) | No |
| **223-231** | **AMDEGYFAY** | **0.02** | **HLA-A*01:01**  **HLA-A*30:02**  **HLA-B*15:01**  **HLA-B*35:01**  **HLA-A*32:01**  **HLA-A*26:01**  **HLA-A*02:06** | **0.7221 (Yes)** | **(No)** | **No** |
| **226-234** | **EGYFAYSHL** | **0.55** | **HLA-B*51:01** | **0.8162 (Yes)** | **(No)** | **No** |
| 229-237 | FAYSHLEKI | 0.02 | HLA-B*51:01  HLA-A*02:06  HLA-A*02:03  HLA-A*68:02  HLA-A*02:01  HLA-B*53:01  HLA-B*08:01 | 0.2999 (No) | (Yes) | No |
| 238-246 | GSCSRGVSK | 0.27 | HLA-A*11:01  HLA-A*30:01  HLA-A*03:01 | -0.0556 (No) | (No) | No |
| **240-248** | **CSRGVSKQR** | **0.46** | **HLA-A*31:01**  **HLA-A*33:01** | **0.7586 (Yes)** | **(No)** | **No** |
| **244-252** | **VSKQRIIGV** | **0.31** | **HLA-A*30:01**  **HLA-B*08:01** | **0.7018 (Yes)** | **(No)** | **No** |
| **248-256** | **RIIGVGEVL** | **0.3** | **HLA-A*32:01**  **HLA-A*02:06**  **HLA-B*15:01**  **HLA-B*07:02** | **0.7453 (Yes)** | **(No)** | **No** |
| **254-262** | **EVLDRGDEV** | **0.1** | **HLA-A*68:02**  **HLA-A*26:01** | **0.7411 (Yes)** | **(No)** | **No** |
| 258-266 | RGDEVPSLF | 0.43 | HLA-B*58:01  HLA-A*01:01  HLA-A*32:01  HLA-B*53:01  HLA-A*30:02  HLA-A*24:02  HLA-A*23:01 | 0.8052 (Yes) | (Yes) | No |
| 262-270 | VPSLFMTNV | 0.03 | HLA-B*51:01  HLA-B*07:02  HLA-B*53:01 | 0.3324 (No) | (No) | No |
| 263-271 | PSLFMTNVW | 0.31 | HLA-B*58:01  HLA-B*57:01 | -0.1700 (No) | (No) | No |
| 271-279 | WTPSNPNTV | 0.43 | HLA-A*68:02 | 0.5066 (Yes) | (Yes) | No |
| 272-280 | TPSNPNTVY | 0.01 | HLA-B*35:01  HLA-B*53:01  HLA-A*26:01  HLA-B*07:02  HLA-A*01:01 | 0.2074 (No) | (Yes) | No |
| 277-285 | NTVYHCSAV | 0.24 | HLA-A*68:02 | 0.1927 (No) | (No) | No |
| 278-286 | TVYHCSAVY | 0.11 | HLA-A*26:01  HLA-A*30:02  HLA-B*15:01  HLA-B*35:01  HLA-A*01:01 | -0.1822 (No) | (No) | No |
| 283-291 | SAVYNSEFY | 0.1 | HLA-B*35:01  HLA-A*26:01  HLA-A*30:02  HLA-A*01:01  HLA-B*53:01  HLA-B*15:01 | 0.2606 (No) | (No) | No |
| **284-292** | **AVYNSEFYY** | **0.01** | **HLA-A*30:02**  **HLA-A*11:01**  **HLA-A*03:01**  **HLA-A*32:01**  **HLA-A*26:01**  **HLA-B*15:01**  **HLA-A*01:01**  **HLA-A*30:01**  **HLA-B*35:01**  **HLA-B*58:01**  **HLA-A*68:01**  **HLA-B*57:01** | **0.4606 (Yes)** | **(No)** | **No** |
| 285-293 | VYNSEFYYV | 0.06 | HLA-A*24:02  HLA-A*23:01 | 0.5496 (Yes) | (Yes) | No |
| **292-300** | **YVLCAVSVV** | **0.77** | **HLA-A*02:06** | **0.8167 (Yes)** | **(No)** | **No** |
| 302-310 | DPILNSTYW | 0.01 | HLA-B*53:01  HLA-B*35:01  HLA-B*51:01 | 0.5142 (Yes) | (Yes) | No |
| 307-315 | STYWSGSLM | 0.21 | HLA-A*26:01  HLA-A*68:02  HLA-A*32:01  HLA-A*30:02 | -0.2933 (No) | (Yes) | No |
| 308-316 | TYWSGSLMM | 0.1 | HLA-A*23:01  HLA-A*24:02 | -0.1591 (No) | (Yes) | No |
| **310-318** | **WSGSLMMTR** | **0.87** | **HLA-A*68:01** | **0.4875 (Yes)** | **(No)** | **No** |
| **313-321** | **SLMMTRLAV** | **0.49** | **HLA-A*02:03**  **HLA-B*08:01**  **HLA-A*02:01** | **0.7233 (Yes)** | **(No)** | **No** |
| **314-322** | **LMMTRLAVK** | **0.31** | **HLA-A*03:01**  **HLA-A*30:01** | **0.8643 (Yes)** | **(No)** | **No** |
| **316-324** | **MTRLAVKPK** | **0.03** | **HLA-A*30:01**  **HLA-A*03:01** | **1.6597 (Yes)** | **(No)** | **No** |
| **322-330** | **KPKNNGESY** | **0.12** | **HLA-B*35:01**  **HLA-B*07:02**  **HLA-B*53:01**  **HLA-A*30:02**  **HLA-B*15:01** | **0.7028 (Yes)** | **(No)** | **No** |
| 327-335 | GESYNQHQF | 0.02 | HLA-B*44:02  HLA-B*44:03  HLA-B*40:01 | 0.2862 (No) | (Yes) | No |
| 328-336 | ESYNQHQFA | 0.26 | HLA-A*68:02 | 0.3246 (No) | (Yes) | No |
| 329-337 | SYNQHQFAL | 0.07 | HLA-A*24:02  HLA-A*23:01  HLA-B*08:01 | 0.5548 (Yes) | (Yes) | No |
| **330-338** | **YNQHQFALR** | **0.21** | **HLA-A*33:01**  **HLA-A*31:01** | **0.6897 (Yes)** | **(No)** | **No** |
| 340-348 | IEKGMYDKV | 0.73 | HLA-B*40:01 | -0.2141 (No) | (No) | No |
| **343-351** | **GMYDKVMPY** | **0.01** | **HLA-B*15:01**  **HLA-A*30:02**  **HLA-A*03:01**  **HLA-A*32:01**  **HLA-A*26:01**  **HLA-A*11:01**  **HLA-A*30:01**  **HLA-A*01:01**  **HLA-B*35:01**  **HLA-A*02:03** | **0.6838 (Yes)** | **(No)** | **No** |
| 347-355 | KVMPYGPSG | 0.8 | HLA-A*30:01 | 0.7825 (Yes) | (Yes) | No |
| 348-356 | VMPYGPSGI | 0.83 | HLA-A*02:03 | 0.5878 (Yes) | (Yes) | No |
| 349-357 | MPYGPSGIK | 1 | HLA-A*68:01 | 1.1937 (Yes) | (Yes) | No |
| **355-363** | **GIKQGDTLY** | **0.03** | **HLA-B*15:01**  **HLA-A*30:02**  **HLA-A*26:01**  **HLA-A*01:01** | **0.8665 (Yes)** | **(No)** | **No** |
| **361-369** | **TLYFPAVGF** | **0.13** | **HLA-A*32:01**  **HLA-B*15:01**  **HLA-A*26:01**  **HLA-A*23:01** | **0.7663 (Yes)** | **(No)** | **No** |
| **362-370** | **LYFPAVGFL** | **0.09** | **HLA-A*23:01**  **HLA-A*24:02** | **0.8143 (Yes)** | **(No)** | **No** |
| **363-371** | **YFPAVGFLV** | **0.39** | **HLA-A*24:02**  **HLA-A*23:01** | **0.8129 (Yes)** | **(No)** | **No** |
| 364-372 | FPAVGFLVR | 0.75 | HLA-A*33:01  HLA-B*35:01 | 1.2033 (Yes) | (Yes) | No |
| 367-375 | VGFLVRTEF | 0.94 | HLA-B*15:01 | 1.8351 (Yes) | (Yes) | No |
| 368-376 | GFLVRTEFK | 0.96 | HLA-A*30:01 | 1.6705 (Yes) | (Yes) | No |
| 369-377 | FLVRTEFKY | 0.33 | HLA-A*30:02  HLA-B*15:01  HLA-A*01:01  HLA-B*35:01  HLA-A*26:01 | 1.4892 (Yes) | (Yes) | No |
| 388-396 | QYSKPENCR | 0.27 | HLA-A*33:01  HLA-A*31:01 | -0.9632 (No) | (Yes) | No |
| 391-399 | KPENCRLSM | 0.04 | HLA-B*07:02  HLA-B*35:01 | -0.2929 (No) | (No) | No |
| **399-407** | **MGIRPNSHY** | **0.14** | **HLA-A*30:02**  **HLA-B*15:01**  **HLA-B*35:01**  **HLA-B*58:01**  **HLA-B*53:01**  **HLA-A*01:01**  **HLA-A*26:01** | **0.8710 (Yes)** | **(No)** | **No** |
| 400-408 | GIRPNSHYI | 0.24 | HLA-A*30:01  HLA-A*02:03  HLA-A*32:01 | 0.3995 (No) | (Yes) | No |
| 402-410 | RPNSHYILR | 0.36 | HLA-A*33:01  HLA-A*31:01 | -0.2222 (No) | (Yes) | No |
| 406-414 | HYILRSGLL | 0.33 | HLA-A*24:02  HLA-A*23:01  HLA-B*08:01 | 0.2102 (No) | (No) | No |
| 407-415 | YILRSGLLK | 0.12 | HLA-A*03:01  HLA-A*11:01  HLA-A*30:01 | -0.7594 (No) | (Yes) | No |
| 408-416 | ILRSGLLKY | 0.02 | HLA-B*15:01  HLA-A*30:02  HLA-A*03:01  HLA-A*32:01  HLA-A*30:01  HLA-A*01:01  HLA-A*26:01 | -0.8478 (No) | (No) | No |
| 410-418 | RSGLLKYNL | 0.62 | HLA-A*32:01  HLA-B*58:01  HLA-B*57:01 | -0.0970 (No) | (No) | No |
| 418-426 | LSDEENSKI | 0.62 | HLA-A*01:01 | 0.6591 (Yes) | (Yes) | No |
| 420-428 | DEENSKIIF | 0.29 | HLA-B*44:03  HLA-B*44:02  HLA-B*40:01 | 0.5190 (Yes) | (Yes) | No |
| **421-429** | **EENSKIIFI** | **0.05** | **HLA-B*44:02**  **HLA-B*44:03**  **HLA-B*40:01** | **0.7042 (Yes)** | **(No)** | **No** |
| 423-431 | NSKIIFIEI | 0.57 | HLA-B*51:01  HLA-B*08:01  HLA-A*68:02 | 0.9375 (Yes) | (Yes) | No |
| 427-435 | IFIEISDQR | 0.13 | HLA-A*33:01  HLA-A*31:01 | 1.0994 (Yes) | (Yes) | No |
| 430-438 | EISDQRLSI | 0.12 | HLA-A*68:02  HLA-A*26:01 | 1.2708 (Yes) | (Yes) | No |
| **435-443** | **RLSIGSPSK** | **0.02** | **HLA-A*03:01**  **HLA-A*30:01**  **HLA-A*11:01** | **0.7713 (Yes)** | **(No)** | **No** |
| **436-444** | **LSIGSPSKI** | **0.72** | **HLA-B*51:01**  **HLA-B*58:01** | **0.8445 (Yes)** | **(No)** | **No** |
| 437-445 | SIGSPSKIY | 0.08 | HLA-A*30:02  HLA-B*15:01  HLA-A*26:01  HLA-A*01:01 | 0.1052 (No) | (Yes) | No |
| 440-448 | SPSKIYDSL | 0.04 | HLA-B*07:02  HLA-B*53:01  HLA-B*35:01  HLA-B*08:01  HLA-B*51:01 | -0.3318 (No) | (Yes) | No |
| 444-452 | IYDSLGQPV | 0.77 | HLA-A*24:02 | -0.5893 (No) | (No) | No |
| 445-453 | YDSLGQPVF | 0.96 | HLA-B*40:01 | -0.2684 (No) | (No) | No |
| 446-454 | DSLGQPVFY | 0.1 | HLA-A*26:01  HLA-A*01:01  HLA-B*35:01  HLA-B*53:01  HLA-A*30:02 | -0.2743 (No) | (Yes) | No |
| **450-458** | **QPVFYQASF** | **0.05** | **HLA-B*35:01**  **HLA-B*53:01**  **HLA-B*07:02**  **HLA-B*51:01** | **0.4601 (Yes)** | **(No)** | **No** |
| **452-460** | **VFYQASFSW** | **0.02** | **HLA-A*23:01**  **HLA-A*24:02**  **HLA-A*32:01**  **HLA-B*58:01**  **HLA-B*57:01**  **HLA-B*53:01** | **1.0092 (Yes)** | **(No)** | **No** |
| **455-463** | **QASFSWDTM** | **0.7** | **HLA-B*35:01** | **0.8540 (Yes)** | **(No)** | **No** |
| **456-464** | **ASFSWDTMI** | **0.64** | **HLA-A*32:01** | **0.5630 (Yes)** | **(No)** | **No** |
| **458-466** | **FSWDTMIKF** | **0.1** | **HLA-B*58:01**  **HLA-B*57:01**  **HLA-B*53:01**  **HLA-A*26:01**  **HLA-A*32:01**  **HLA-B*35:01**  **HLA-A*23:01**  **HLA-B*15:01**  **HLA-A*02:06**  **HLA-A*24:02**  **HLA-B*51:01** | **0.4980 (Yes)** | **(No)** | **No** |
| **461-469** | **DTMIKFGDV** | **0.93** | **HLA-A*68:02** | **1.3974 (Yes)** | **(No)** | **No** |
| **467-475** | **GDVQTVNPL** | **0.78** | **HLA-B*40:01** | **0.6447 (Yes)** | **(No)** | **No** |
| 468-476 | DVQTVNPLV | 0.12 | HLA-A*68:02  HLA-B*51:01 | 0.6735 (Yes) | (Yes) | No |
| 471-479 | TVNPLVVNW | 0.01 | HLA-A*32:01  HLA-B*58:01  HLA-B*57:01  HLA-B*53:01  HLA-A*26:01  HLA-A*23:01  HLA-A*24:02  HLA-B*44:02  HLA-B*44:03  HLA-B*35:01 | 0.9223 (Yes) | (Yes) | No |
| 506-514 | GVYNDAFLI | 0.49 | HLA-A*02:06  HLA-A*32:01  HLA-A*02:01 | 0.0788 (No) | (Yes) | No |
| **511-519** | **AFLIDRINW** | **0.22** | **HLA-A*23:01**  **HLA-A*24:02**  **HLA-A*32:01**  **HLA-B*58:01**  **HLA-B*57:01** | **0.9509 (Yes)** | **(No)** | **No** |
| 512-520 | FLIDRINWI | 0.02 | HLA-A*02:03  HLA-A*02:01  HLA-A*02:06  HLA-B*08:01 | 0.7847 (Yes) | (Yes) | No |
| 516-524 | RINWISAGV | 0.32 | HLA-A*02:03  HLA-A*32:01  HLA-A*02:06  HLA-A*02:01 | 2.1854 (Yes) | (Yes) | No |
| **518-526** | **NWISAGVFL** | **0.55** | **HLA-A*23:01**  **HLA-A*24:02** | **0.7512 (Yes)** | **(No)** | **No** |
| 529-537 | NQTAENPVF | 0.83 | HLA-B*15:01 | 0.3117 (No) | (Yes) | No |
| 530-538 | QTAENPVFT | 0.31 | HLA-A*68:02 | 0.3061 (No) | (No) | No |
| 531-539 | TAENPVFTV | 0.25 | HLA-A*68:02  HLA-B*51:01  HLA-A*02:06 | 0.3876 (No) | (No) | No |
| 532-540 | AENPVFTVF | 0.01 | HLA-B*44:02  HLA-B*44:03  HLA-B*40:01  HLA-B*15:01  HLA-A*26:01  HLA-A*23:01  HLA-B*35:01  HLA-B*53:01  HLA-A*24:02  HLA-A*32:01  HLA-A*30:02 | 0.0979 (No) | (Yes) | No |
| 537-545 | FTVFKDNEV | 0.14 | HLA-A*68:02  HLA-A*02:06 | 0.0167 (No) | (Yes) | No |
| 538-546 | TVFKDNEVL | 0.54 | HLA-A*68:02  HLA-B*35:01 | 0.0637 (No) | (Yes) | No |
| 539-547 | VFKDNEVLY | 0.06 | HLA-A*30:02  HLA-A*23:01  HLA-A*24:02  HLA-B*15:01  HLA-B*35:01  HLA-A*26:01  HLA-A*01:01 | 0.0622 (No) | (Yes) | No |
| 543-551 | NEVLYRAQL | 0.15 | HLA-B*40:01  HLA-B*44:03  HLA-B*44:02 | -0.0969 (No) | (Yes) | No |
| 544-552 | EVLYRAQLA | 0.94 | HLA-A*68:02 | 0.3924 (No) | (No) | No |
| 552-560 | ASEDTNAQK | 0.33 | HLA-A*11:01 | 0.2337 (No) | (Yes) | No |
| 558-566 | AQKTITNCF | 0.01 | HLA-B*15:01  HLA-B*44:02  HLA-A*32:01  HLA-B*44:03  HLA-A*30:02  HLA-A*24:02  HLA-A*23:01 | 0.0837 (No) | (Yes) | No |
| 560-568 | KTITNCFLL | 0.38 | HLA-A*32:01  HLA-B*58:01  HLA-B*57:01 | 0.2452 (No) | (No) | No |
| 561-569 | TITNCFLLK | 0.21 | HLA-A*11:01  HLA-A*03:01 | -0.0797 (No) | (Yes) | No |
| 564-572 | NCFLLKNKI | 0.96 | HLA-B*51:01 | 0.7885 (Yes) | (Yes) | No |
| 567-575 | LLKNKIWCI | 0.08 | HLA-B*08:01  HLA-A*02:03  HLA-A*02:01 | 1.6610 (Yes) | (Yes) | No |
| **572-580** | **IWCISLVEI** | **0.61** | **HLA-A*24:02**  **HLA-A*23:01** | **1.7382 (Yes)** | **(No)** | **No** |
| 573-581 | WCISLVEIY | 0.53 | HLA-B*35:01 | 1.4886 (Yes) | (Yes) | No |
| 579-587 | EIYDTGDNV | 0.05 | HLA-A*68:02  HLA-A*26:01 | 0.3280 (No) | (Yes) | No |
| 580-588 | IYDTGDNVI | 0.52 | HLA-A*24:02  HLA-A*23:01 | -0.1922 (No) | (Yes) | No |
| 583-591 | TGDNVIRPK | 0.98 | HLA-A*11:01 | 0.3311 (No) | (Yes) | No |
| 584-592 | GDNVIRPKL | 1 | HLA-B*40:01 | -0.0725 (No) | (Yes) | No |
| 586-594 | NVIRPKLFA | 0.45 | HLA-A*68:02 | -0.1698 (No) | (Yes) | No |
| **587-595** | **VIRPKLFAV** | **0.05** | **HLA-B*08:01**  **HLA-A*02:03**  **HLA-A*30:01**  **HLA-A*02:06**  **HLA-A*02:01**  **HLA-A*32:01**  **HLA-B*07:02** | **0.7478 (Yes)** | **(No)** | **No** |
| **589-597** | **RPKLFAVKI** | **0.07** | **HLA-B*07:02**  **HLA-B*51:01**  **HLA-B*08:01** | **0.5410 (Yes)** | **(No)** | **No** |

(Probable ANTIGEN: Yes; Probable NON-ANTIGEN: No; Probable ALLERGEN: Yes, Probable NON-ALLERGEN: No  )

**Supplementary Table 7.** Identified MHC II epitope within the Glycoprotein, along with other properties.

| Position | Sequence | P. rank | Allele | Antigen | Allergen | Toxic |
| --- | --- | --- | --- | --- | --- | --- |
| **5-19** | **SKKVRFENTASDKGK** | **6.8** | **HLA-DRB1*03:01**  **HLA-DRB1*07:01**  **HLA-DRB1*15:01**  **HLA-DRB3*01:01**  **HLA-DRB3*02:02**  **HLA-DRB4*01:01**  **HLA-DRB5*01:01** | **1.0749 (Yes)** | **No** | **No** |
| **21-35** | **PSKVIKSYYGTMDIK** | **1.4** | **HLA-DRB1*15:01** | **0.5721 (Yes)** | **No** | **No** |
| **26-40** | **KSYYGTMDIKKINEG** | **13** | **HLA-DRB1*07:01**  **HLA-DRB3*02:02**  **HLA-DRB5*01:01** | **0.8235 (Yes)** | **No** | **No** |
| **31-45** | **TMDIKKINEGLLDSK** | **12** | **HLA-DRB1*07:01**  **HLA-DRB1*15:01**  **HLA-DRB3*02:02**  **HLA-DRB4*01:01**  **HLA-DRB5*01:01** | **1.0167 (Yes)** | **No** | **No** |
| 37-51 | INEGLLDSKILSAFN | 5.1 | HLA-DRB1*03:01  HLA-DRB4*01:01 | 0.4780 (Yes) | Yes | No |
| **42-56** | **LDSKILSAFNTVIAL** | **9.7** | **HLA-DRB1*07:01**  **HLA-DRB1*15:01** | **0.4034 (Yes)** | **No** | **No** |
| **64-78** | **VMNIMIIQNYTRSTD** | **8.5** | **HLA-DRB1*03:01**  **HLA-DRB1*15:01**  **HLA-DRB3*01:01**  **HLA-DRB3*02:02**  **HLA-DRB4*01:01**  **HLA-DRB5*01:01** | **0.5664 (Yes)** | **No** | **No** |
| 70-84 | IQNYTRSTDNQAMIK | 4 | HLA-DRB1*07:01  HLA-DRB3*01:01  HLA-DRB3*02:02  HLA-DRB4*01:01  HLA-DRB5*01:01 | 0.2122 (No) | Yes | No |
| 79-93 | NQAMIKDALQSIQQQ | 3.4 | HLA-DRB1*03:01  HLA-DRB3*01:01  HLA-DRB3*02:02  HLA-DRB4*01:01 | 0.1918 (No) | No | No |
| 87-101 | LQSIQQQIKGLADKI | 10 | HLA-DRB1*03:01  HLA-DRB4*01:01 | -0.0317 (No) | No | No |
| 92-106 | QQIKGLADKIGTEIG | 17 | HLA-DRB1*15:01  HLA-DRB4*01:01 | 0.0807 (No) | Yes | No |
| 98-112 | ADKIGTEIGPKVSLI | 14 | HLA-DRB1*15:01  HLA-DRB3*01:01  HLA-DRB3*02:02 | 0.6620 (Yes) | Yes | No |
| **104-118** | **EIGPKVSLIDTSSTI** | **20** | **HLA-DRB3*01:01**  **HLA-DRB4*01:01** | **1.1984 (Yes)** | **No** | **No** |
| 109-123 | VSLIDTSSTITIPAN | 18 | HLA-DRB1*03:01  HLA-DRB1*07:01  HLA-DRB3*01:01  HLA-DRB3*02:02 | 0.4993 (Yes) | Yes | No |
| **123-137** | **NIGLLGSKISQSTAS** | **7** | **HLA-DRB4*01:01** | **1.0711 (Yes)** | **No** | **No** |
| 128-142 | GSKISQSTASINENV | 7 | HLA-DRB1*07:01  HLA-DRB3*02:02  HLA-DRB4*01:01 | 0.1275 (No) | No | No |
| 135-149 | TASINENVNEKCKFT | 17 | HLA-DRB1*03:01  HLA-DRB3*01:01  HLA-DRB3*02:02 | 0.5146 (Yes) | Yes | No |
| 164-178 | NPLPFREYKPQTEGV | 12 | HLA-DRB1*07:01  HLA-DRB1*15:01  HLA-DRB5*01:01 | 0.8804 (Yes) | Yes | No |
| 169-183 | REYKPQTEGVSNLVG | 19 | HLA-DRB5*01:01 | 0.3602 (No) | Yes | No |
| 178-192 | VSNLVGLPNNICLQK | 3.5 | HLA-DRB1*15:01  HLA-DRB3*02:02  HLA-DRB4*01:01  HLA-DRB5*01:01 | 0.2508 (No) | Yes | No |
| **185-199** | **PNNICLQKTSNQILK** | **3.5** | **HLA-DRB1*07:01**  **HLA-DRB4*01:01** | **0.4568 (Yes)** | **No** | **No** |
| **199-213** | **KPKLISYTLPVVGQS** | **5** | **HLA-DRB1*07:01**  **HLA-DRB1*15:01**  **HLA-DRB3*02:02**  **HLA-DRB4*01:01**  **HLA-DRB5*01:01** | **0.7131 (Yes)** | **No** | **No** |
| 213-227 | SGTCITDPLLAMDEG | 18 | HLA-DRB1*03:01  HLA-DRB3*01:01 | 0.2595 (No) | No | No |
| **219-233** | **DPLLAMDEGYFAYSH** | **3.3** | **HLA-DRB1*03:01**  **HLA-DRB1*15:01**  **HLA-DRB3*01:01**  **HLA-DRB3*02:02** | **0.5979 (Yes)** | **No** | **No** |
| **225-239** | **DEGYFAYSHLEKIGS** | **0.82** | **HLA-DRB1*07:01**  **HLA-DRB1*15:01**  **HLA-DRB3*01:01**  **HLA-DRB3*02:02**  **HLA-DRB4*01:01**  **HLA-DRB5*01:01** | **0.4582 (Yes)** | **No** | **No** |
| **241-255** | **SRGVSKQRIIGVGEV** | **3.4** | **HLA-DRB4*01:01** | **1.0135 (Yes)** | **No** | **No** |
| 247- | QRIIGVGEVLDRGDE | 7 | HLA-DRB1*03:01  HLA-DRB4*01:01  HLA-DRB5*01:01 | 0.7420 (Yes) | Yes | No |
| 252-266 | VGEVLDRGDEVPSLF | 12 | HLA-DRB3*01:01 | 0.8189 (Yes) | Yes | No |
| 262-276 | VPSLFMTNVWTPSNP | 6.7 | HLA-DRB3*02:02 | -0.2903 (No) | No | No |
| 267-281 | MTNVWTPSNPNTVYH | 0.96 | HLA-DRB1*07:01  HLA-DRB3*01:01  HLA-DRB3*02:02  HLA-DRB5*01:01 | -0.2685 (No) | Yes | No |
| 275-289 | NPNTVYHCSAVYNSE | 16 | HLA-DRB1*07:01  HLA-DRB3*02:02 | 0.1549 (No) | Yes | No |
| 289-303 | EFYYVLCAVSVVGDP | 18 | HLA-DRB1*07:01  HLA-DRB3*01:01  HLA-DRB3*02:02 | 0.4618 (Yes) | Yes | No |
| 295-309 | CAVSVVGDPILNSTY | 1.5 | HLA-DRB1*03:01  HLA-DRB3*01:01  HLA-DRB3*02:02  HLA-DRB4*01:01 | 0.3150 (No) | Yes | No |
| **311-325** | **SGSLMMTRLAVKPKN** | **11** | **HLA-DRB5*01:01** | **1.3059 (Yes)** | **No** | **No** |
| **316-330** | **MTRLAVKPKNNGESY** | **20** | **HLA-DRB1*03:01**  **HLA-DRB4*01:01** | **1.0965 (Yes)** | **No** | **No** |
| 334-348 | QFALRNIEKGMYDKV | 5.4 | HLA-DRB1*03:01 | 0.7976 (Yes) | Yes | No |
| 340-354 | IEKGMYDKVMPYGPS | 5.3 | HLA-DRB1*03:01  HLA-DRB3*01:01 | 0.3067 (No) | No | No |
| **345-359** | **YDKVMPYGPSGIKQG** | **13** | **HLA-DRB1*07:01**  **HLA-DRB1*15:01** | **0.8397 (Yes)** | **No** | **No** |
| 353-367 | PSGIKQGDTLYFPAV | 9 | HLA-DRB1*03:01  HLA-DRB1*07:01  HLA-DRB1*15:01  HLA-DRB3*01:01  HLA-DRB4*01:01 | 0.4035 (Yes) | Yes | No |
| **362-376** | **LYFPAVGFLVRTEFK** | **17** | **HLA-DRB1*07:01** | **1.2567 (Yes)** | **No** | **No** |
| 367-381 | VGFLVRTEFKYNDSN | 14 | HLA-DRB1*03:01 | 1.4978 (Yes) | Yes | No |
| 372-386 | RTEFKYNDSNCPVAK | 11 | HLA-DRB3*01:01  HLA-DRB3*02:02  HLA-DRB5*01:01 | 0.8220 (Yes) | Yes | No |
| 398-412 | SMGIRPNSHYILRSG | 3.5 | HLA-DRB1*03:01  HLA-DRB1*15:01  HLA-DRB3*01:01  HLA-DRB3*02:02  HLA-DRB4*01:01 | 0.7152 (Yes) | Yes | No |
| 405-419 | SHYILRSGLLKYNLS | 18 | HLA-DRB1*03:01  HLA-DRB5*01:01 | -0.0036 (No) | Yes | No |
| 411-425 | SGLLKYNLSDEENSK | 12 | HLA-DRB1*03:01  HLA-DRB3*01:01  HLA-DRB3*02:02  HLA-DRB4*01:01 | 0.4851 (Yes) | Yes | No |
| 423-437 | NSKIIFIEISDQRLS | 3.2 | HLA-DRB1*03:01  HLA-DRB1*07:01  HLA-DRB1*15:01  HLA-DRB3*01:01  HLA-DRB3*02:02  HLA-DRB4*01:01  HLA-DRB5*01:01 | 1.1278 (Yes) | Yes | No |
| **428-442** | **FIEISDQRLSIGSPS** | **15** | **HLA-DRB1*03:01**  **HLA-DRB4*01:01** | **1.1599 (Yes)** | **No** | **No** |
| 433-447 | DQRLSIGSPSKIYDS | 11 | HLA-DRB1*07:01  HLA-DRB3*02:02 | 0.5365 (Yes) | Yes | No |
| 441-445 | PSKIYDSLGQPVFYQ | 1.9 | HLA-DRB1*07:01  HLA-DRB1*15:01  HLA-DRB3*01:01  HLA-DRB3*02:02 | -0.2723 (No) | Yes | No |
| **448-462** | **LGQPVFYQASFSWDT** | **16** | **HLA-DRB1*07:01**  **HLA-DRB1*15:01** | **0.4393 (Yes)** | **No** | **No** |
| **455-469** | **QASFSWDTMIKFGDV** | **8** | **HLA-DRB1*03:01**  **HLA-DRB3*01:01**  **HLA-DRB3*02:02** | **1.1926 (Yes)** | **No** | **No** |
| **460-474** | **WDTMIKFGDVQTVNP** | **11** | **HLA-DRB1*07:01**  **HLA-DRB1*15:01**  **HLA-DRB3*01:01**  **HLA-DRB3*02:02**  **HLA-DRB4*01:01** | **1.0247 (Yes)** | **No** | **No** |
| 467-481 | GDVQTVNPLVVNWRD | 12 | HLA-DRB1*07:01  HLA-DRB3*02:02  HLA-DRB5*01:01 | 1.1968 (Yes) | Yes | No |
| 472-486 | VNPLVVNWRDNTVIS | 6.6 | HLA-DRB1*03:01  HLA-DRB1*15:01  HLA-DRB3*02:02  HLA-DRB4*01:01 | 1.2409 (Yes) | Yes | No |
| **477-491** | **VNWRDNTVISRPGQS** | **14** | **HLA-DRB1*03:01**  **HLA-DRB3*01:01**  **HLA-DRB3*02:02**  **HLA-DRB5*01:01** | **0.7659 (Yes)** | **No** | **No** |
| 504-518 | WEGVYNDAFLIDRIN | 14 | HLA-DRB3*01:01 | 0.0122 (No) | No | No |
| 509-523 | NDAFLIDRINWISAG | 3.8 | HLA-DRB1*03:01  HLA-DRB1*07:01  HLA-DRB3*01:01  HLA-DRB3*02:02  HLA-DRB4*01:01  HLA-DRB5*01:01 | 1.0310 (Yes) | Yes | No |
| **514-528** | **IDRINWISAGVFLDS** | **7.3** | **HLA-DRB1*07:01**  **HLA-DRB3*01:01** | **0.6199 (Yes)** | **No** | **No** |
| 521-535 | SAGVFLDSNQTAENP | 0.93 | HLA-DRB1*03:01  HLA-DRB1*15:01  HLA-DRB3*01:01  HLA-DRB3*02:02  HLA-DRB4*01:01 | 0.2228 (No) | No | No |
| 531-545 | TAENPVFTVFKDNEV | 4.1 | HLA-DRB1*07:01  HLA-DRB1*15:01  HLA-DRB3*01:01 | 0.2052 (No) | Yes | No |
| 536-550 | VFTVFKDNEVLYRAQ | 1.2 | HLA-DRB1*03:01  HLA-DRB1*15:01  HLA-DRB3*01:01  HLA-DRB3*02:02  HLA-DRB5*01:01 | 0.0492 (No) | No | No |
| 542-556 | DNEVLYRAQLASEDT | 17 | HLA-DRB1*07:01  HLA-DRB1*15:01  HLA-DRB3*02:02  HLA-DRB4*01:01  HLA-DRB5*01:01 | 0.2639 (No) | Yes | No |
| **548-562** | **RAQLASEDTNAQKTI** | **5.8** | **HLA-DRB1*03:01**  **HLA-DRB1*15:01**  **HLA-DRB3*01:01**  **HLA-DRB3*02:02**  **HLA-DRB4*01:01**  **HLA-DRB5*01:01** | **0.5408 (Yes)** | **No** | **No** |
| 577-591 | LVEIYDTGDNVIRPK | 3.7 | HLA-DRB1*07:01  HLA-DRB1*15:01  HLA-DRB3*01:01  HLA-DRB3*02:02  HLA-DRB5*01:01 | 0.3801 (No) | Yes | No |
| 588-602 | IRPKLFAVKIPEQCT | 16 | HLA-DRB3*02:02  HLA-DRB4*01:01  HLA-DRB5*01:01 | 0.5349 (Yes) | Yes | No |

(Probable ANTIGEN: Yes; Probable NON-ANTIGEN: No; Probable ALLERGEN: Yes, Probable NON-ALLERGEN: No  )

**Supplementary Table 8.** Identified MHC I epitope within the fusion protein, along with other properties.

| **Position** | **Peptide** | **Percentile rank** | **Allele** | **Antigen** | **Allergen** | **Toxic** |
| --- | --- | --- | --- | --- | --- | --- |
| **1-9** | **MAVILNKRY** | **0.12** | **HLA-B*35:01**  **HLA-B*53:01**  **HLA-A*26:01**  **HLA-B*58:01**  **HLA-A*30:02**  **HLA-B*57:01**  **HLA-B*15:01** | **1.5818 (Yes)** | **No** | **No** |
| 2-10 | AVILNKRYY | 0.11 | HLA-A*30:02  HLA-A*26:01  HLA-B*15:01  HLA-A*32:01 | 1.4578 (Yes) | Yes | No |
| **5-13** | **LNKRYYSNL** | **0.67** | **HLA-B*08:01** | **0.4451 (Yes)** | **No** | **No** |
| 8-16 | RYYSNLLLL | 0.01 | HLA-A*23:01  HLA-A*24:02  HLA-A*30:01  HLA-A*32:01  HLA-A*30:02 | 0.2157 (No) | Yes | No |
| 9-17 | YYSNLLLLI | 0.03 | HLA-A*24:02  HLA-A*23:01 | 0.4777 (Yes) | Yes | No |
| **10-18** | **YSNLLLLIL** | **0.98** | **HLA-B*58:01** | **0.4488 (Yes)** | **No** | **No** |
| 17-25 | ILMISECSV | 0.57 | HLA-A*02:01  HLA-A*02:03 | -0.2208 (No) | Yes | No |
| 19-27 | MISECSVGI | 0.54 | HLA-A*68:02 | 0.0711 (No) | Yes | No |
| **21-29** | **SECSVGILH** | **0.48** | **HLA-B*44:03**  **HLA-B*44:02** | **0.5473 (Yes)** | **No** | **No** |
| 22-30 | ECSVGILHY | 0.33 | HLA-A*26:01  HLA-B*35:01  HLA-A*01:01  HLA-B*53:01 | 0.8418 (Yes) | Yes | No |
| **24-32** | **SVGILHYEK** | **0.19** | **HLA-A*11:01**  **HLA-A*03:01**  **HLA-A*30:01**  **HLA-A*68:01** | **1.6328 (Yes)** | **No** | **No** |
| **25-33** | **VGILHYEKL** | **0.85** | **HLA-B*08:01** | **1.4183 (Yes)** | **No** | **No** |
| 27-35 | ILHYEKLSK | 0.14 | HLA-A*03:01  HLA-A*30:01  HLA-A*11:01 | 0.2942 (No) | No | No |
| 30-38 | YEKLSKIGL | 0.08 | HLA-B*40:01  HLA-B*44:02  HLA-B*44:03  HLA-B*08:01 | 1.0137 (Yes) | Yes | No |
| **32-40** | **KLSKIGLVK** | **0.02** | **HLA-A*03:01**  **HLA-A*30:01**  **HLA-A*11:01** | **0.5490 (Yes)** | **No** | **No** |
| 36-44 | IGLVKGITR | 0.72 | HLA-A*31:01 | 0.0703 (No) | Yes | No |
| 37-45 | GLVKGITRK | 0.05 | HLA-A*03:01  HLA-A*11:01 | -0.5235 (No) | No | No |
| 38-46 | LVKGITRKY | 0.06 | HLA-A*26:01  HLA-A*30:02  HLA-B*15:01  HLA-A*32:01  HLA-A*30:01  HLA-B*57:01  HLA-B*35:01 | -0.2545 (No) | No | No |
| **42-50** | **ITRKYKIKS** | **0.99** | **HLA-A*30:01** | **0.6494 (Yes)** | **No** | **No** |
| **45-53** | **KYKIKSNPL** | **0.41** | **HLA-A*24:02**  **HLA-B*08:01**  **HLA-A*23:01**  **HLA-A*30:01** | **1.3144 (Yes)** | **No** | **No** |
| **47-55** | **KIKSNPLTK** | **0.01** | **HLA-A*30:01**  **HLA-A*03:01**  **HLA-A*11:01**  **HLA-A*31:01** | **0.7250 (Yes)** | **No** | **No** |
| 49-57 | KSNPLTKDI | 0.58 | HLA-B*58:01  HLA-B*57:01 | 0.3292 (No) | No | No |
| 51-59 | NPLTKDIVI | 0.07 | HLA-B*51:01  HLA-B*08:01  HLA-B*53:01  HLA-B*35:01  HLA-B*07:02 | -0.0340 (No) | Yes | No |
| 53-61 | LTKDIVIKM | 0.08 | HLA-A*26:01  HLA-A*68:02  HLA-B*57:01  HLA-A*30:01  HLA-A*32:01  HLA-B*58:01  HLA-B*15:01 | -0.4565 (No) | No | No |
| 57-65 | IVIKMIPNV | 0.05 | HLA-A*02:06  HLA-A*02:01  HLA-A*02:03  HLA-A*68:02  HLA-B*51:01  HLA-A*32:01 | 0.4739 (Yes) | Yes | No |
| 60-68 | KMIPNVSNM | 0.09 | HLA-B*15:01  HLA-A*32:01  HLA-A*02:03  HLA-A*02:06  HLA-A*02:01  HLA-A*30:02  HLA-A*30:01  HLA-A*23:01  HLA-A*24:02  HLA-A*26:01 | 0.3542 (No) | No | No |
| 71-79 | CTGSVMENY | 0.26 | HLA-A*01:01  HLA-A*26:01  HLA-A*30:02 | 0.2761 (No) | Yes | No |
| **74-82** | **SVMENYKTR** | **0.07** | **HLA-A*68:01**  **HLA-A*31:01**  **HLA-A*33:01**  **HLA-A*11:01**  **HLA-A*30:01**  **HLA-A*03:01**  **HLA-A*26:01** | **0.4955 (Yes)** | **No** | **No** |
| **78-86** | **NYKTRLNGI** | **0.76** | **HLA-A*24:02**  **HLA-B*08:01** | **0.4371 (Yes)** | **No** | **No** |
| 80-88 | KTRLNGILT | 0.14 | HLA-A*30:01 | 0.2660 (No) | No | No |
| 82-90 | RLNGILTPI | 0.06 | HLA-A*02:03  HLA-A*32:01  HLA-A*02:01  HLA-A*02:06 | -0.1321 (No) | No | No |
| 86-94 | ILTPIKGAL | 0.67 | HLA-A*02:03 | -0.1887 (No) | Yes | No |
| 88-96 | TPIKGALEI | 0.03 | HLA-B*51:01  HLA-B*53:01  HLA-B*07:02  HLA-B*35:01 | 0.2655 (No) | No | No |
| 89-97 | PIKGALEIY | 0.79 | HLA-B*15:01  HLA-A*30:02 | 0.2933 (No) | Yes | No |
| 96-104 | IYKNNTHDL | 0.08 | HLA-A*24:02  HLA-A*23:01  HLA-B*08:01 | 0.2457 (No) | No | No |
| 100-108 | NTHDLVGDV | 0.25 | HLA-A*68:02 | 0.1176 (No) | No | No |
| 107-115 | DVRLAGVIM | 0.47 | HLA-A*26:01 | 0.8235 (Yes) | Yes | No |
| **114-122** | **IMAGVAIGI** | **0.28** | **HLA-A*02:03**  **HLA-A*02:01**  **HLA-A*32:01**  **HLA-A*02:06** | **1.0244 (Yes)** | **No** | **No** |
| **117-125** | **GVAIGIATA** | **0.52** | **HLA-A*02:03**  **HLA-A*02:06** | **0.9956 (Yes)** | **No** | **No** |
| **118-126** | **VAIGIATAA** | **0.98** | **HLA-A*68:02** | **1.2240 (Yes)** | **No** | **No** |
| **120-128** | **IGIATAAQI** | **0.75** | **HLA-B*51:01** | **1.0247 (Yes)** | **No** | **No** |
| 124-132 | TAAQITAGV | 0.01 | HLA-A*68:02  HLA-B*51:01  HLA-A*02:06 | 0.8057 (Yes) | Yes | No |
| **126-134** | **AQITAGVAL** | **0.06** | **HLA-B*15:01**  **HLA-A*02:06**  **HLA-B*40:01** | **0.7995 (Yes)** | **No** | **No** |
| **127-135** | **QITAGVALY** | **0.07** | **HLA-A*26:01**  **HLA-A*30:02**  **HLA-B*15:01**  **HLA-B*35:01**  **HLA-A*01:01** | **0.6567 (Yes)** | **No** | **No** |
| 131-139 | GVALYEAMK | 0.56 | HLA-A*11:01  HLA-A*03:01 | 0.3586 (No) | Yes | No |
| 133-141 | ALYEAMKNA | 0.02 | HLA-A*02:03  HLA-A*02:01  HLA-A*02:06 | -0.1101 (No) | Yes | No |
| 136-144 | EAMKNADNI | 0.38 | HLA-B*51:01  HLA-A*68:02 | 0.3335 (No) | No | No |
| 143-151 | NINKLKSSI | 0.53 | HLA-B*08:01 | -0.4582 (No) | No | No |
| **146-154** | **KLKSSIEST** | **0.31** | **HLA-A*02:03**  **HLA-A*30:01** | **0.7429 (Yes)** | **No** | **No** |
| 149-157 | SSIESTNEA | 0.38 | HLA-A*68:02  HLA-A*02:06 | 1.0500 (Yes) | Yes | No |
| 151-159 | IESTNEAVV | 0.45 | HLA-B*40:01 | 0.7207 (Yes) | Yes | No |
| 152-160 | ESTNEAVVK | 0.45 | HLA-A*68:01 | -0.0464 (No) | Yes | No |
| 153-161 | STNEAVVKL | 0.04 | HLA-A*68:02  HLA-A*02:06  HLA-A*32:01  HLA-A*26:01  HLA-A*02:03  HLA-A*02:01  HLA-A*30:01  HLA-B*58:01 | -0.1397 (No) | No | No |
| **157-165** | **AVVKLQETA** | **0.87** | **HLA-A*02:06** | **1.0926 (Yes)** | **No** | **No** |
| 162-170 | QETAEKTVY | 0.02 | HLA-B*44:02  HLA-B*44:03  HLA-B*40:01 | -0.0307 (No) | No | No |
| 163-171 | ETAEKTVYV | 0.01 | HLA-A*68:02  HLA-A*26:01  HLA-A*02:06  HLA-A*02:03  HLA-A*02:01  HLA-B*51:01 | 0.2861 (No) | No | No |
| 164-172 | TAEKTVYVL | 0.59 | HLA-B*35:01  HLA-B*51:01  HLA-B*53:01 | 0.3815 (No) | No | No |
| **167-175** | **KTVYVLTAL** | **0.12** | **HLA-A*32:01**  **HLA-A*02:06**  **HLA-A*68:02**  **HLA-B*58:01**  **HLA-B*57:01**  **HLA-A*30:01** | **0.4890 (Yes)** | **No** | **No** |
| **170-178** | **YVLTALQDY** | **0.24** | **HLA-A*26:01**  **HLA-A*30:02**  **HLA-B*35:01**  **HLA-B*15:01**  **HLA-A*01:01** | **0.4714 (Yes)** | **No** | **No** |
| 175-183 | LQDYINTNL | 0.3 | HLA-A*02:06  HLA-A*02:01  HLA-B*40:01 | -0.0840 (No) | Yes | No |
| **179-187** | **INTNLVPTI** | **0.87** | **HLA-B*51:01** | **0.7834 (Yes)** | **No** | **No** |
| 182-190 | NLVPTIDKI | 0.37 | HLA-A*02:06  HLA-A*02:01  HLA-A*02:03  HLA-A*68:02 | 0.4572 (Yes) | Yes | No |
| 185-193 | PTIDKISCK | 0.79 | HLA-A*11:01  HLA-A*68:01 | 0.5501 (Yes) | Yes | No |
| 189-197 | KISCKQTEL | 0.84 | HLA-B*08:01 | 1.8648 (Yes) | Yes | No |
| 191-199 | SCKQTELSL | 0.71 | HLA-B*08:01 | 1.5654 (Yes) | Yes | No |
| 193-201 | KQTELSLDL | 0.32 | HLA-A*02:06 | 1.5855 (Yes) | Yes | No |
| **195-203** | **TELSLDLAL** | **0.05** | **HLA-B*40:01**  **HLA-B*44:03**  **HLA-B*44:02** | **1.1768 (Yes)** | **No** | **No** |
| 197-205 | LSLDLALSK | 0.1 | HLA-A*11:01  HLA-A*03:01  HLA-A*30:01 | 0.8937 (Yes) | Yes | No |
| **198-206** | **SLDLALSKY** | **0.02** | **HLA-A*01:01**  **HLA-A*30:02**  **HLA-A*26:01**  **HLA-B*15:01** | **0.8317 (Yes)** | **No** | **No** |
| 202-210 | ALSKYLSDL | 0.29 | HLA-A*02:03  HLA-A*02:01 | -0.3438 (No) | No | No |
| 205-213 | KYLSDLLFV | 0.23 | HLA-A*23:01  HLA-A*24:02 | 0.4039 (Yes) | Yes | No |
| **206-214** | **YLSDLLFVF** | **0.15** | **HLA-A*32:01**  **HLA-A*23:01**  **HLA-B*15:01**  **HLA-A*24:02**  **HLA-A*02:06**  **HLA-A*02:01**  **HLA-B*35:01**  **HLA-B*53:01**  **HLA-A*26:01**  **HLA-B*58:01**  **HLA-A*02:03** | **0.6851 (Yes)** | **No** | **No** |
| 210-218 | LLFVFGPNL | 0.36 | HLA-A*02:01  HLA-A*02:03  HLA-A*02:06  HLA-A*32:01 | 0.0890 (No) | No | No |
| 218-226 | LQDPVSNSM | 0.1 | HLA-A*02:06  HLA-B*15:01  HLA-B*35:01  HLA-B*40:01  HLA-A*02:01  HLA-A*01:01  HLA-B*08:01  HLA-A*30:02  HLA-B*53:01 | -0.3774 (No) | No | No |
| 220-228 | DPVSNSMTI | 0.03 | HLA-B*51:01  HLA-B*53:01  HLA-B*35:01 | 0.4141 (Yes) | Yes | No |
| 226-234 | MTIQAISQA | 0.05 | HLA-A*68:02  HLA-A*02:06  HLA-A*26:01 | 0.8906 (Yes) | Yes | No |
| 227-235 | TIQAISQAF | 0.16 | HLA-B*15:01  HLA-B*35:01  HLA-A*26:01  HLA-A*32:01  HLA-B*53:01 | 0.4295 (Yes) | Yes | No |
| 231-239 | ISQAFGGNY | 0.12 | HLA-A*30:02  HLA-A*01:01  HLA-B*15:01 | -0.2649 (No) | Yes | No |
| 234-242 | AFGGNYETL | 0.38 | HLA-A*24:02  HLA-A*23:01 | 0.0102 (No) | No | No |
| 238-246 | NYETLLRTL | 0.18 | HLA-A*24:02  HLA-A*23:01  HLA-B*08:01 | -0.5424 (No) | Yes | No |
| 240-248 | ETLLRTLGY | 0.03 | HLA-A*26:01  HLA-A*01:01  HLA-A*30:02  HLA-B*35:01  HLA-A*68:01 | -0.4652 (No) | No | No |
| 241-249 | TLLRTLGYA | 0.94 | HLA-A*02:03 | -0.0590 (No) | No | No |
| 249-257 | ATEDFDDLL | 0.53 | HLA-A*01:01 | 0.3337 (No) | Yes | No |
| 258-266 | ESDSITGQI | 0.44 | HLA-A*68:02  HLA-A*01:01 | 0.2486 (No) | Yes | No |
| 260-268 | DSITGQIIY | 0.07 | HLA-A*26:01  HLA-B*35:01  HLA-A*01:01  HLA-B*53:01 | 0.2130 (No) | No | No |
| 261-269 | SITGQIIYV | 0.05 | HLA-A*02:06  HLA-A*02:01  HLA-A*68:02  HLA-A*02:03 | 0.5185 (Yes) | Yes | No |
| 266-274 | IIYVDLSGY | 0.19 | HLA-A*30:02  HLA-B*15:01  HLA-A*26:01  HLA-B*35:01 | 0.1452 (No) | No | No |
| 267-275 | IYVDLSGYY | 0.11 | HLA-A*30:02  HLA-A*23:01  HLA-A*24:02  HLA-A*26:01 | -0.1694 (No) | Yes | No |
| 268-276 | YVDLSGYYI | 0.31 | HLA-A*02:06  HLA-A*01:01  HLA-A*02:01  HLA-A*68:02 | 0.2966 (No) | Yes | No |
| 270-278 | DLSGYYIIV | 0.92 | HLA-A*68:02 | -0.1130 (No) | Yes | No |
| 272-280 | SGYYIIVRV | 0.48 | HLA-A*68:02  HLA-B*51:01  HLA-A*02:06 | -0.1152 (No) | Yes | No |
| 273-281 | GYYIIVRVY | 0.13 | HLA-A*30:02  HLA-A*23:01  HLA-A*24:02 | -0.1971 (No) | No | No |
| 274-282 | YYIIVRVYF | 0.03 | HLA-A*23:01  HLA-A*24:02 | 0.2201 (No) | Yes | No |
| 277-285 | IVRVYFPIL | 0.78 | HLA-B*08:01 | 0.1537 (No) | No | No |
| 279-287 | RVYFPILTE | 0.43 | HLA-A*30:01  HLA-A*03:01 | 0.3346 (No) | Yes | No |
| 280-288 | VYFPILTEI | 0.01 | HLA-A*23:01  HLA-A*24:02  HLA-A*32:01  HLA-B*51:01 | 0.3454 (No) | Yes | No |
| 282-290 | FPILTEIQQ | 0.43 | HLA-B*35:01 | 0.6981 (Yes) | Yes | No |
| 284-292 | ILTEIQQAY | 0.02 | HLA-B*15:01  HLA-A*30:02  HLA-B*35:01  HLA-A*01:01  HLA-A*26:01  HLA-A*32:01  HLA-B*53:01 | 0.3843 (No) | Yes | No |
| 288-296 | IQQAYIQEL | 0.08 | HLA-A*02:06  HLA-A*02:01  HLA-B*15:01  HLA-A*32:01  HLA-A*02:03  HLA-B*40:01 | 0.1002 (No) | No | No |
| 289-297 | QQAYIQELL | 0.46 | HLA-A*02:06  HLA-B*40:01  HLA-B*15:01 | -0.2494 (No) | No | No |
| 291-299 | AYIQELLPV | 0.52 | HLA-A*24:02  HLA-A*23:01 | 0.3982 (No) | No | No |
| **293-301** | **IQELLPVSF** | **0.18** | **HLA-B*15:01**  **HLA-A*32:01**  **HLA-A*23:01**  **HLA-A*24:02** | **0.9776 (Yes)** | **No** | **No** |
| 300-308 | SFNNDNSEW | 0.48 | HLA-A*24:02  HLA-B*58:01  HLA-A*23:01  HLA-B*53:01  HLA-B*57:01 | 0.3715 (No) | No | No |
| **304-312** | **DNSEWISIV** | **0.66** | **HLA-B*51:01** | **0.9030 (Yes)** | **No** | **No** |
| 307-315 | EWISIVPNF | 0.03 | HLA-A*23:01  HLA-A*24:02  HLA-A*26:01 | 1.0607 (Yes) | Yes | No |
| **309-317** | **ISIVPNFIL** | **0.5** | **HLA-B*58:01**  **HLA-B*57:01** | **0.7808 (Yes)** | **No** | **No** |
| **310-318** | **SIVPNFILV** | **0.03** | **HLA-A*02:06**  **HLA-A*68:02**  **HLA-A*02:03**  **HLA-A*02:01**  **HLA-A*26:01** | **0.5759 (Yes)** | **No** | **No** |
| 311-319 | IVPNFILVR | 0.34 | HLA-A*68:01  HLA-A*31:01  HLA-A*33:01  HLA-A*11:01 | 0.2796 (No) | Yes | No |
| 314-322 | NFILVRNTL | 0.34 | HLA-B*08:01  HLA-A*23:01  HLA-A*24:02 | 0.4368 (Yes) | Yes | No |
| **315-323** | **FILVRNTLI** | **0.56** | **HLA-B*08:01** | **0.5200 (Yes)** | **No** | **No** |
| 320-328 | NTLISNIEI | 0.65 | HLA-A*68:02 | 0.4056 (Yes) | Yes | No |
| 322-330 | LISNIEIGF | 0.63 | HLA-A*32:01 | 1.9750 (Yes) | Yes | No |
| 327-335 | EIGFCLITK | 0.79 | HLA-A*68:01 | 1.3384 (Yes) | Yes | No |
| 328-336 | IGFCLITKR | 0.93 | HLA-A*31:01 | 2.1929 (Yes) | Yes | No |
| 331-339 | CLITKRSVI | 0.49 | HLA-B*08:01 | 1.3230 (Yes) | Yes | No |
| 336-344 | RSVICNQDY | 0.58 | HLA-A*30:02  HLA-B*58:01 | 0.3440 (No) | No | No |
| 344-352 | YATPMTNNM | 0.09 | HLA-B*35:01  HLA-B*53:01  HLA-B*51:01  HLA-A*68:02  HLA-A*26:01 | 0.2930 (No) | No | No |
| 345-353 | ATPMTNNMR | 0.64 | HLA-A*68:01 | 0.6130 (Yes) | Yes | No |
| 360-368 | TEKCPRELV | 0.9 | HLA-B*40:01 | -0.2766 (No) | Yes | No |
| 365-373 | RELVVSSHV | 0.25 | HLA-B*40:01  HLA-B*44:02  HLA-B*44:03 | 0.2035 (No) | No | No |
| 367-375 | LVVSSHVPR | 0.12 | HLA-A*68:01  HLA-A*31:01  HLA-A*33:01  HLA-A*11:01 | -0.3252 (No) | Yes | No |
| 368-376 | VVSSHVPRF | 0.06 | HLA-A*32:01  HLA-A*26:01  HLA-A*23:01  HLA-B*15:01  HLA-A*24:02  HLA-B*58:01  HLA-A*30:02  HLA-B*57:01  HLA-B*53:01  HLA-B*35:01 | -0.6689 (No) | Yes | No |
| 370-378 | SSHVPRFAL | 0.29 | HLA-B*07:02  HLA-A*30:01  HLA-A*32:01  HLA-B*08:01 | -0.6148 (No) | No | No |
| 376-384 | FALSNGVLF | 0.08 | HLA-B*35:01  HLA-B*53:01  HLA-B*58:01  HLA-B*51:01  HLA-A*23:01  HLA-B*57:01  HLA-A*24:02  HLA-B*15:01 | 0.2380 (No) | No | No |
| 377-385 | ALSNGVLFA | 0.21 | HLA-A*02:03  HLA-A*02:01  HLA-A*02:06 | 0.1191 (No) | No | No |
| 382-390 | VLFANCISV | 0.17 | HLA-A*02:03  HLA-A*02:01  HLA-A*02:06 | -0.0008 (No) | Yes | No |
| 401-409 | ISQSGEQTL | 0.69 | HLA-B*58:01 | 0.2919 (No) | Yes | No |
| 402-410 | SQSGEQTLL | 0.42 | HLA-A*02:06  HLA-B*15:01  HLA-B*40:01 | 0.1692 (No) | No | No |
| 403-411 | QSGEQTLLM | 0.84 | HLA-A*01:01 | 0.1148 (No) | No | No |
| **414-422** | **NTTCPTAVL** | **0.37** | **HLA-A*68:02** | **0.4763 (Yes)** | **No** | **No** |
| 417-425 | CPTAVLGNV | 0.61 | HLA-B*51:01 | 0.1167 (No) | No | No |
| 419-427 | TAVLGNVII | 0.49 | HLA-B*51:01 | -0.1201 (No) | Yes | No |
| 421-429 | VLGNVIISL | 0.04 | HLA-A*02:01  HLA-A*02:03  HLA-A*02:06  HLA-A*32:01 | 0.2971 (No) | Yes | No |
| **424-432** | **NVIISLGKY** | **0.03** | **HLA-A*26:01**  **HLA-A*30:02**  **HLA-B*35:01**  **HLA-B*15:01** | **0.6683 (Yes)** | **No** | **No** |
| 428-436 | SLGKYLGSV | 0.07 | HLA-A*02:03  HLA-A*02:01  HLA-A*02:06 | -0.3809 (No) | No | No |
| **430-438** | **GKYLGSVNY** | **0.39** | **HLA-A*30:02**  **HLA-B*15:01** | **0.4493 (Yes)** | **No** | **No** |
| **435-443** | **SVNYNSEGI** | **0.56** | **HLA-A*68:02** | **0.7710 (Yes)** | **No** | **No** |
| 437-445 | NYNSEGIAI | 0.4 | HLA-A*24:02  HLA-A*23:01 | 0.9515 (Yes) | Yes | No |
| **441-449** | **EGIAIGPPV** | **0.35** | **HLA-A*68:02**  **HLA-B*51:01** | **0.6599 (Yes)** | **No** | **No** |
| **442-450** | **GIAIGPPVF** | **0.31** | **HLA-B*15:01**  **HLA-A*32:01** | **0.7334 (Yes)** | **No** | **No** |
| 446-454 | GPPVFTDKV | 0.63 | HLA-B*51:01 | 0.6976 (Yes) | Yes | No |
| 450-458 | FTDKVDISS | 0.67 | HLA-A*01:01 | 0.5729 (Yes) | Yes | No |
| 455-463 | DISSQISSM | 0.05 | HLA-A*26:01  HLA-B*35:01  HLA-A*68:02  HLA-B*08:01 | 0.5778 (Yes) | Yes | No |
| 459-467 | QISSMNQSL | 0.72 | HLA-A*68:02 | 0.5806 (Yes) | Yes | No |
| 465-473 | QSLQQSKDY | 0.54 | HLA-A*30:02  HLA-A*01:01 | 0.5968 (Yes) | Yes | No |
| 472-480 | DYIKEAQRL | 0.14 | HLA-A*24:02  HLA-A*23:01 | 0.2024 (No) | Yes | No |
| 473-481 | YIKEAQRLL | 0.24 | HLA-B*08:01  HLA-A*02:03  HLA-A*26:01 | 0.0983 (No) | No | No |
| 476-484 | EAQRLLDTV | 0.28 | HLA-B*51:01  HLA-A*68:02 | -0.0109 (No) | Yes | No |
| **480-488** | **LLDTVNPSL** | **0.02** | **HLA-A*02:01**  **HLA-A*02:06**  **HLA-A*02:03**  **HLA-B*08:01** | **0.5529 (Yes)** | **No** | **No** |
| 483-491 | TVNPSLISM | 0.06 | HLA-A*26:01  HLA-A*68:02  HLA-B*35:01  HLA-A*02:06  HLA-A*32:01  HLA-B*15:01  HLA-B*07:02  HLA-A*30:02  HLA-B*53:01  HLA-A*30:01  HLA-A*02:03 | 1.2454 (Yes) | Yes | No |
| **487-495** | **SLISMLSMI** | **0.13** | **HLA-A*02:03**  **HLA-A*02:01**  **HLA-A*02:06** | **0.4599 (Yes)** | **No** | **No** |
| **490-498** | **SMLSMIILY** | **0.06** | **HLA-A*30:02**  **HLA-A*32:01**  **HLA-B*15:01**  **HLA-A*03:01**  **HLA-A*11:01**  **HLA-A*26:01**  **HLA-A*01:01** | **0.4005 (Yes)** | **No** | **No** |
| **491-499** | **MLSMIILYV** | **0.23** | **HLA-A*02:01**  **HLA-A*02:03**  **HLA-A*02:06** | **0.4460 (Yes)** | **No** | **No** |
| **492-500** | **LSMIILYVL** | **0.96** | **HLA-B*58:01** | **0.4751 (Yes)** | **No** | **No** |
| 497-505 | LYVLSIASL | 0.16 | HLA-A*23:01  HLA-A*24:02 | 0.7392 (Yes) | Yes | No |
| **501-509** | **SIASLCIGL** | **0.81** | **HLA-A*68:02**  **HLA-A*02:06** | **1.5870 (Yes)** | **No** | **No** |
| **504-512** | **SLCIGLITF** | **0.22** | **HLA-A*32:01**  **HLA-B*15:01**  **HLA-A*23:01**  **HLA-A*24:02** | **1.4681 (Yes)** | **No** | **No** |
| **507-515** | **IGLITFISF** | **0.87** | **HLA-A*23:01** | **1.2089 (Yes)** | **No** | **No** |
| 508-516 | GLITFISFI | 0.13 | HLA-A*02:03  HLA-A*02:01  HLA-A*02:06 | 0.5153 (Yes) | Yes | No |
| **512-520** | **FISFIIVEK** | **0.12** | **HLA-A*68:01**  **HLA-A*11:01**  **HLA-A*03:01** | **1.1861 (Yes)** | **No** | **No** |
| **513-521** | **ISFIIVEKK** | **0.24** | **HLA-A*11:01**  **HLA-A*03:01**  **HLA-A*68:01**  **HLA-A*30:01** | **2.3237 (Yes)** | **No** | **No** |
| **514-522** | **SFIIVEKKR** | **0.14** | **HLA-A*33:01**  **HLA-A*31:01** | **2.3755 (Yes)** | **No** | **No** |
| **517-525** | **IVEKKRNTY** | **0.15** | **HLA-A*30:02**  **HLA-A*01:01**  **HLA-B*15:01**  **HLA-A*32:01** | **1.5454 (Yes)** | **No** | **No** |
| 519-527 | EKKRNTYSR | 0.84 | HLA-A*33:01 | -0.0890 (No) | No | No |
| 523-531 | NTYSRLEDR | 0.08 | HLA-A*33:01  HLA-A*68:01  HLA-A*31:01 | 1.0814 (Yes) | Yes | No |
| **524-532** | **TYSRLEDRR** | **0.8** | **HLA-A*33:01** | **0.7886 (Yes)** | **No** | **No** |
| 534-542 | RPTSSGDLY | 0.13 | HLA-B*35:01  HLA-B*53:01  HLA-A*01:01  HLA-A*30:02 | 0.5682 (Yes) | Yes | No |
| 535-543 | PTSSGDLYY | 0.03 | HLA-A*01:01  HLA-A*30:02 | 0.5061 (Yes) | Yes | No |
| 536-544 | TSSGDLYYI | 0.32 | HLA-A*68:02  HLA-B*58:01 | 0.4062 (Yes) | Yes | No |

(Probable ANTIGEN: Yes; Probable NON-ANTIGEN: No; Probable ALLERGEN: Yes, Probable NON-ALLERGEN: No  )

**Supplementary Table 9.** Identified MHC II epitope within the fusion protein, along with other properties.

| Position | Peptide sequence | P. rank | Allele | Antigen | Allergen | Toxic |
| --- | --- | --- | --- | --- | --- | --- |
| 1-15 | MAVILNKRYYSNLLL | 19 | HLA-DRB1*03:01 | 0.7185 (Yes) | Yes | No |
| **6-20** | **NKRYYSNLLLLILMI** | **19** | **HLA-DRB3*01:01**  **HLA-DRB3*02:02** | **0.4145 (Yes)** | **No** | **No** |
| **23-37** | **CSVGILHYEKLSKIG** | **15** | **HLA-DRB1*03:01**  **HLA-DRB1*15:01**  **HLA-DRB4*01:01** | **1.1148 (Yes)** | **No** | **No** |
| 33-47 | LSKIGLVKGITRKYK | 12 | HLA-DRB1*03:01  HLA-DRB1*15:01  HLA-DRB5*01:01 | 0.1296 (No) | No | No |
| 38-52 | LVKGITRKYKIKSNP | 19 | HLA-DRB1*03:01  HLA-DRB4*01:01 | 0.3378 (No) | No | No |
| **45-59** | **KYKIKSNPLTKDIVI** | **7.5** | **HLA-DRB1*03:01**  **HLA-DRB3*01:01**  **HLA-DRB3*02:02**  **HLA-DRB4*01:01** | **0.6237 (Yes)** | **No** | **No** |
| 50-64 | SNPLTKDIVIKMIPN | 1.1 | HLA-DRB1*03:01  HLA-DRB3*01:01  HLA-DRB3*02:02  HLA-DRB4*01:01 | -0.1842 (No) | No | No |
| **56-70** | **DIVIKMIPNVSNMSQ** | **17** | **HLA-DRB1*03:01**  **HLA-DRB1*07:01**  **HLA-DRB1*15:01**  **HLA-DRB3*02:02**  **HLA-DRB4*01:01**  **HLA-DRB5*01:01** | **0.5776 (Yes)** | **No** | **No** |
| 71-85 | CTGSVMENYKTRLNG | 6.5 | HLA-DRB1*03:01  HLA-DRB1*15:01  HLA-DRB3*02:02 | 0.0665 (No) | No | No |
| 87-101 | LTPIKGALEIYKNNT | 6.4 | HLA-DRB5*01:01 | 0.1945 (No) | No | No |
| 93-107 | ALEIYKNNTHDLVGD | 8.7 | HLA-DRB1*03:01  HLA-DRB1*07:01  HLA-DRB1*15:01  HLA-DRB3*01:01  HLA-DRB3*02:02  HLA-DRB4*01:01  HLA-DRB5*01:01 | 0.0989 (No) | Yes | No |
| **100-114** | **NTHDLVGDVRLAGVI** | **0.23** | **HLA-DRB1*03:01**  **HLA-DRB3*01:01**  **HLA-DRB3*02:02**  **HLA-DRB4*01:01** | **0.4952 (Yes)** | **No** | **No** |
| **105-119** | **VGDVRLAGVIMAGVA** | **6.3** | **HLA-DRB4*01:01** | **0.6663 (Yes)** | **No** | **No** |
| **111-125** | **AGVIMAGVAIGIATA** | **12** | **HLA-DRB1*07:01**  **HLA-DRB1*15:01**  **HLA-DRB3*02:02** | **0.6963 (Yes)** | **No** | **No** |
| **117-131** | **GVAIGIATAAQITAG** | **17** | **HLA-DRB1*03:01**  **HLA-DRB1*07:01**  **HLA-DRB1*15:01**  **HLA-DRB3*01:01**  **HLA-DRB3*02:02**  **HLA-DRB4*01:01**  **HLA-DRB5*01:01** | **1.0404 (Yes)** | **No** | **No** |
| 131-145 | GVALYEAMKNADNIN | 15 | HLA-DRB1*15:01  HLA-DRB3*02:02  HLA-DRB5*01:01 | 0.5354 (Yes) | Yes | No |
| 136-150 | EAMKNADNINKLKSS | 15 | HLA-DRB3*01:01  HLA-DRB5*01:01 | 0.0503 (No) | Yes | No |
| 141-155 | ADNINKLKSSIESTN | 20 | HLA-DRB1*07:01  HLA-DRB1*15:01  HLA-DRB5*01:01 | 0.2442 (No) | Yes | No |
| 148-162 | KSSIESTNEAVVKLQ | 1.8 | HLA-DRB1*07:01  HLA-DRB1*15:01  HLA-DRB3*01:01  HLA-DRB3*02:02 | 0.6772 (Yes) | Yes | No |
| 155-169 | NEAVVKLQETAEKTV | 19 | HLA-DRB1*03:01  HLA-DRB4*01:01  HLA-DRB5*01:01 | 0.3495 (No) | Yes | No |
| 166-180 | EKTVYVLTALQDYIN | 14 | HLA-DRB1*07:01  HLA-DRB1*15:01  HLA-DRB3*01:01  HLA-DRB3*02:02  HLA-DRB4*01:01  HLA-DRB5*01:01 | 0.2183 (No) | No | No |
| 171-185 | VLTALQDYINTNLVP | 1.4 | HLA-DRB1*15:01 | 0.3874 (No) | No | No |
| 176-190 | QDYINTNLVPTIDKI | 7 | HLA-DRB1*03:01  HLA-DRB1*07:01  HLA-DRB3*01:01  HLA-DRB3*02:02  HLA-DRB4*01:01 | 0.2939 (No) | Yes | No |
| **187-201** | **IDKISCKQTELSLDL** | **15** | **HLA-DRB4*01:01** | **1.1451 (Yes)** | **No** | **No** |
| 194-208 | QTELSLDLALSKYLS | 2.3 | HLA-DRB1*03:01  HLA-DRB3*01:01  HLA-DRB5*01:01 | 0.6172 (Yes) | Yes | No |
| 203-217 | LSKYLSDLLFVFGPN | 3.6 | HLA-DRB3*01:01 | 0.0133 (No) | No | No |
| 208-222 | SDLLFVFGPNLQDPV | 14 | HLA-DRB1*07:01  HLA-DRB1*15:01  HLA-DRB3*01:01  HLA-DRB3*02:02  HLA-DRB4*01:01  HLA-DRB5*01:01 | 0.3912 (No) | Yes | No |
| 219-233 | QDPVSNSMTIQAISQ | 18 | HLA-DRB1*07:01  HLA-DRB4*01:01 | 0.3475 (No) | Yes | No |
| 224-238 | NSMTIQAISQAFGGN | 0.57 | HLA-DRB4*01:01 | 0.2019 (No) | Yes | No |
| 231-245 | ISQAFGGNYETLLRT | 13 | HLA-DRB3*01:01  HLA-DRB3*02:02 | -0.3655 (No) | No | No |
| 236-250 | GGNYETLLRTLGYAT | 16 | HLA-DRB5*01:01 | -0.0986 (No) | Yes | No |
| 254-268 | DDLLESDSITGQIIY | 4.4 | HLA-DRB1*03:01  HLA-DRB3*01:01 | 0.0181 (No) | Yes | No |
| 263-277 | TGQIIYVDLSGYYII | 2.1 | HLA-DRB1*03:01  HLA-DRB3*01:01 | 0.2737 (No) | Yes | No |
| 276-290 | IIVRVYFPILTEIQQ | 12 | HLA-DRB1*15:01 | 0.3580 (No) | No | No |
| 281-295 | YFPILTEIQQAYIQE | 7.1 | HLA-DRB1*03:01  HLA-DRB3*01:01 | 0.4791 (Yes) | Yes | No |
| **286-300** | **TEIQQAYIQELLPVS** | **17** | **HLA-DRB4*01:01** | **0.5068 (Yes)** | **No** | **No** |
| 298-312 | PVSFNNDNSEWISIV | 8.8 | HLA-DRB1*03:01  HLA-DRB1*07:01  HLA-DRB3*01:01  HLA-DRB3*02:02 | 0.9155 (Yes) | Yes | No |
| **307-321** | **EWISIVPNFILVRNT** | **4.2** | **HLA-DRB1*03:01**  **HLA-DRB1*07:01**  **HLA-DRB1*15:01**  **HLA-DRB3*01:01**  **HLA-DRB3*02:02** | **0.8280 (Yes)** | **No** | **No** |
| 313-327 | PNFILVRNTLISNIE | 2.2 | HLA-DRB1*07:01  HLA-DRB1*15:01  HLA-DRB3*01:01  HLA-DRB3*02:02  HLA-DRB5*01:01 | 0.6480 (Yes) | Yes | No |
| 335-349 | KRSVICNQDYATPMT | 2.7 | HLA-DRB3*02:02 | 0.6157 (Yes) | Yes | No |
| 340-354 | CNQDYATPMTNNMRE | 16 | HLA-DRB3*02:02 | 0.2340 (No) | Yes | No |
| 363-377 | CPRELVVSSHVPRFA | 1.5 | HLA-DRB1*03:01  HLA-DRB1*07:01  HLA-DRB3*01:01  HLA-DRB3*02:02  HLA-DRB4*01:01  HLA-DRB5*01:01 | -0.2167 (No) | Yes | No |
| 372-386 | HVPRFALSNGVLFAN | 0.57 | HLA-DRB1*07:01  HLA-DRB3*01:01  HLA-DRB3*02:02  HLA-DRB5*01:01 | -0.4069 (No) | No | No |
| 398-412 | GRAISQSGEQTLLMI | 15 | HLA-DRB1*07:01 | 0.3377 (No) | No | No |
| 407-421 | QTLLMIDNTTCPTAV | 18 | HLA-DRB1*03:01  HLA-DRB3*02:02 | 0.5169 (Yes) | Yes | No |
| 418-432 | PTAVLGNVIISLGKY | 3.9 | HLA-DRB3*02:02 | 0.3024 (No) | Yes | No |
| 423-437 | GNVIISLGKYLGSVN | 9.4 | HLA-DRB1*03:01  HLA-DRB1*07:01  HLA-DRB1*15:01  HLA-DRB4*01:01  HLA-DRB5*01:01 | 0.2757 (No) | No | No |
| 428-442 | SLGKYLGSVNYNSEG | 7 | HLA-DRB1*07:01  HLA-DRB3*02:02  HLA-DRB5*01:01 | 0.2614 (No) | Yes | No |
| 433-447 | LGSVNYNSEGIAIGP | 14 | HLA-DRB1*07:01  HLA-DRB3*02:02  HLA-DRB4*01:01 | 1.3932 (Yes) | Yes | No |
| **440-454** | **SEGIAIGPPVFTDKV** | **19** | **HLA-DRB1*15:01**  **HLA-DRB3*02:02**  **HLA-DRB4*01:01** | **0.6188 (Yes)** | **No** | **No** |
| 446-460 | GPPVFTDKVDISSQI | 1.9 | HLA-DRB1*03:01  HLA-DRB3*01:01  HLA-DRB3*02:02  HLA-DRB4*01:01 | 0.6365 (Yes) | Yes | No |
| 451-465 | TDKVDISSQISSMNQ | 7.4 | HLA-DRB4*01:01 | 0.5913 (Yes) | Yes | No |
| 457-471 | SSQISSMNQSLQQSK | 17 | HLA-DRB1*15:01  HLA-DRB4*01:01 | 0.4987 (Yes) | Yes | No |
| 464-478 | NQSLQQSKDYIKEAQ | 19 | HLA-DRB1*03:01  HLA-DRB1*15:01  HLA-DRB5*01:01 | 0.2162 (No) | No | No |
| 471-485 | KDYIKEAQRLLDTVN | 5.3 | HLA-DRB1*03:01  HLA-DRB1*07:01  HLA-DRB3*01:01  HLA-DRB3*02:02  HLA-DRB4*01:01  HLA-DRB5*01:01 | 0.0731 (No) | No | No |
| 476-490 | EAQRLLDTVNPSLIS | 8.8 | HLA-DRB1*07:01 | 0.4912 (Yes) | Yes | No |
| **508-522** | **GLITFISFIIVEKKR** | **7.9** | **HLA-DRB5*01:01** | **1.4212 (Yes)** | **No** | **No** |
| **513-527** | **ISFIIVEKKRNTYSR** | **4.5** | **HLA-DRB1*03:01**  **HLA-DRB3*02:02**  **HLA-DRB4*01:01**  **HLA-DRB5*01:01** | **1.2290 (Yes)** | **No** | **No** |
| **523-537** | **NTYSRLEDRRVRPTS** | **14** | **HLA-DRB1*07:01**  **HLA-DRB1*15:01**  **HLA-DRB5*01:01** | **0.8149 (Yes)** | **No** | **No** |
| 530-544 | DRRVRPTSSGDLYYI | 0.45 | HLA-DRB1*07:01  HLA-DRB1*15:01 | 0.9946 (Yes) | Yes | No |

(Probable ANTIGEN: Yes; Probable NON-ANTIGEN: No; Probable ALLERGEN: Yes, Probable NON-ALLERGEN: No  )

**Supplementary Table 10.** List of vaccine-TLR2 docked complex model with score.

| Cluster | Members | Representative | Weighted Score |
| --- | --- | --- | --- |
| 0 | 78 | Center | -1143.5 |
|  |  | Lowest Energy | -1162.9 |
| 1 | 78 | Center | -993.6 |
|  |  | Lowest Energy | -1068.7 |
| **2** | **53** | **Center** | **-1011.6** |
|  |  | **Lowest Energy** | **-1242.7** |
| 3 | 48 | Center | -1045.6 |
|  |  | Lowest Energy | -1050.5 |
| 4 | 41 | Center | -1068.8 |
|  |  | Lowest Energy | -1111.1 |
| 5 | 39 | Center | -1050.8 |
|  |  | Lowest Energy | -1078.3 |
| 6 | 34 | Center | -940.7 |
|  |  | Lowest Energy | -1082.8 |
| 7 | 32 | Center | -1030.6 |
|  |  | Lowest Energy | -1138.7 |
| 8 | 27 | Center | -1104.8 |
|  |  | Lowest Energy | -1177.7 |
| 9 | 25 | Center | -985.7 |
|  |  | Lowest Energy | -1224.9 |
| 10 | 24 | Center | -953.6 |
|  |  | Lowest Energy | -1038.9 |
| 11 | 22 | Center | -1093.8 |
|  |  | Lowest Energy | -1093.8 |
| 12 | 20 | Center | -934.6 |
|  |  | Lowest Energy | -1090.6 |
| 13 | 18 | Center | -997.3 |
|  |  | Lowest Energy | -1044.7 |
| 14 | 18 | Center | -990.8 |
|  |  | Lowest Energy | -1062.3 |
| 15 | 18 | Center | -984.1 |
|  |  | Lowest Energy | -1120.2 |
| 16 | 17 | Center | -934.6 |
|  |  | Lowest Energy | -1108.0 |
| 17 | 17 | Center | -983.8 |
|  |  | Lowest Energy | -1013.9 |
| 18 | 17 | Center | -983.5 |
|  |  | Lowest Energy | -1052.3 |
| 19 | 17 | Center | -990.3 |
|  |  | Lowest Energy | -1114.7 |
| 20 | 16 | Center | -1010.0 |
|  |  | Lowest Energy | -1010.0 |
| 21 | 15 | Center | -1062.4 |
|  |  | Lowest Energy | -1062.4 |
| 22 | 14 | Center | -961.4 |
|  |  | Lowest Energy | -1030.8 |
| 23 | 14 | Center | -998.4 |
|  |  | Lowest Energy | -1172.1 |
| 24 | 14 | Center | -1087.0 |
|  |  | Lowest Energy | -1087.0 |
| 25 | 13 | Center | -968.2 |
|  |  | Lowest Energy | -1056.3 |
| 26 | 12 | Center | -1037.6 |
|  |  | Lowest Energy | -1039.4 |
| 27 | 12 | Center | -969.4 |
|  |  | Lowest Energy | -1176.2 |
| 28 | 11 | Center | -940.3 |
|  |  | Lowest Energy | -1002.2 |
| 29 | 10 | Center | -945.5 |
|  |  | Lowest Energy | -977.8 |

**Supplementary Table 11.** List of vaccine-TLR3 docked complex model with score.

| **Cluster** | **Members** | **Representative** | **Weighted Score** |
| --- | --- | --- | --- |
| **0** | **58** | **Center** | **-1321.6** |
|  |  | **Lowest Energy** | **-1321.6** |
| 1 | 53 | Center | -931.6 |
|  |  | Lowest Energy | -1086.9 |
| 2 | 36 | Center | -934.3 |
|  |  | Lowest Energy | -1201.7 |
| 3 | 29 | Center | -1019.7 |
|  |  | Lowest Energy | -1075.5 |
| 4 | 24 | Center | -1163.9 |
|  |  | Lowest Energy | -1163.9 |
| 5 | 23 | Center | -1085.2 |
|  |  | Lowest Energy | -1142.0 |
| 6 | 22 | Center | -941.6 |
|  |  | Lowest Energy | -1133.2 |
| 7 | 20 | Center | -938.7 |
|  |  | Lowest Energy | -1022.6 |
| 8 | 20 | Center | -944.3 |
|  |  | Lowest Energy | -1111.3 |
| 9 | 19 | Center | -950.5 |
|  |  | Lowest Energy | -1093.5 |
| 10 | 19 | Center | -994.2 |
|  |  | Lowest Energy | -1110.4 |
| 11 | 18 | Center | -962.0 |
|  |  | Lowest Energy | -1265.9 |
| 12 | 18 | Center | -1025.6 |
|  |  | Lowest Energy | -1025.6 |
| 13 | 18 | Center | -1025.4 |
|  |  | Lowest Energy | -1050.3 |
| 14 | 18 | Center | -937.0 |
|  |  | Lowest Energy | -1085.0 |
| 15 | 17 | Center | -1129.1 |
|  |  | Lowest Energy | -1129.1 |
| 16 | 17 | Center | -932.6 |
|  |  | Lowest Energy | -1021.7 |
| 17 | 16 | Center | -940.0 |
|  |  | Lowest Energy | -1068.9 |
| 18 | 16 | Center | -960.4 |
|  |  | Lowest Energy | -1011.2 |
| 19 | 15 | Center | -1026.6 |
|  |  | Lowest Energy | -1026.6 |
| 20 | 15 | Center | -1022.8 |
|  |  | Lowest Energy | -1022.8 |
| 21 | 14 | Center | -997.0 |
|  |  | Lowest Energy | -997.0 |
| 22 | 13 | Center | -924.2 |
|  |  | Lowest Energy | -1186.4 |
| 23 | 13 | Center | -1078.0 |
|  |  | Lowest Energy | -1078.0 |
| 24 | 13 | Center | -1054.9 |
|  |  | Lowest Energy | -1054.9 |
| 25 | 13 | Center | -977.5 |
|  |  | Lowest Energy | -1025.0 |
| 26 | 13 | Center | -949.1 |
|  |  | Lowest Energy | -1088.4 |
| 27 | 12 | Center | -1031.9 |
|  |  | Lowest Energy | -1078.5 |
| 28 | 10 | Center | -1037.5 |
|  |  | Lowest Energy | -1037.5 |
| 29 | 10 | Center | -1050.8 |
|  |  | Lowest Energy | -1050.8 |

**Supplementary Table 12.** List of vaccine-TLR4 docked complex model with score.

| **Cluster** | **Members** | **Representative** | **Weighted Score** |
| --- | --- | --- | --- |
| 0 | 64 | Center | -974.2 |
|  |  | Lowest Energy | -1181.0 |
| 1 | 42 | Center | -1013.6 |
|  |  | Lowest Energy | -1105.0 |
| 2 | 38 | Center | -964.5 |
|  |  | Lowest Energy | -1163.3 |
| 3 | 32 | Center | -996.3 |
|  |  | Lowest Energy | -1134.6 |
| 4 | 30 | Center | -1037.8 |
|  |  | Lowest Energy | -1088.5 |
| 5 | 29 | Center | -952.3 |
|  |  | Lowest Energy | -1105.5 |
| 6 | 23 | Center | -1079.8 |
|  |  | Lowest Energy | -1082.6 |
| 7 | 23 | Center | -1039.5 |
|  |  | Lowest Energy | -1057.8 |
| 8 | 22 | Center | -955.5 |
|  |  | Lowest Energy | -1043.0 |
| 9 | 21 | Center | -985.3 |
|  |  | Lowest Energy | -1169.0 |
| 10 | 21 | Center | -1022.2 |
|  |  | Lowest Energy | -1159.0 |
| 11 | 20 | Center | -949.4 |
|  |  | Lowest Energy | -1040.4 |
| 12 | 19 | Center | -985.6 |
|  |  | Lowest Energy | -1036.3 |
| 13 | 19 | Center | -993.7 |
|  |  | Lowest Energy | -1076.6 |
| 14 | 19 | Center | -981.9 |
|  |  | Lowest Energy | -1166.0 |
| 15 | 17 | Center | -1033.1 |
|  |  | Lowest Energy | -1065.1 |
| 16 | 16 | Center | -948.6 |
|  |  | Lowest Energy | -1060.7 |
| 17 | 16 | Center | -1045.5 |
|  |  | Lowest Energy | -1045.5 |
| **18** | **15** | **Center** | **-976.8** |
|  |  | **Lowest Energy** | **-1210.4** |
| 19 | 14 | Center | -1078.8 |
|  |  | Lowest Energy | -1078.8 |
| 20 | 14 | Center | -1011.8 |
|  |  | Lowest Energy | -1026.6 |
| 21 | 12 | Center | -1127.6 |
|  |  | Lowest Energy | -1127.6 |
| 22 | 12 | Center | -1089.1 |
|  |  | Lowest Energy | -1089.1 |
| 23 | 11 | Center | -1001.3 |
|  |  | Lowest Energy | -1059.7 |
| 24 | 11 | Center | -972.4 |
|  |  | Lowest Energy | -1035.2 |
| 25 | 11 | Center | -1076.2 |
|  |  | Lowest Energy | -1094.0 |
| 26 | 11 | Center | -995.7 |
|  |  | Lowest Energy | -996.7 |
| 27 | 11 | Center | -1088.9 |
|  |  | Lowest Energy | -1088.9 |
| 28 | 11 | Center | -950.1 |
|  |  | Lowest Energy | -989.0 |
| 29 | 10 | Center | -940.2 |
|  |  | Lowest Energy | -1053.2 |

**Supplementary Table 13.** List of atomic interactions of the TLR3 and vaccine complex at 0 and 100 ns using the COCOMAPS 2.0 server.

| **Properties** | **Values** | |
| --- | --- | --- |
|  | **0 ns** | **100 ns** |
| Number of H-bonds | 18 | 17 |
| Number of Salt-bridges | 2 | 2 |
| Number of CH-O/N bonds | 20 | 18 |
| Number of apolar van der Waals contacts | 30 | 30 |
| Number of CH–π interactions | 6 | 4 |
| Number of cation–π interactions | 0 | 0 |

**
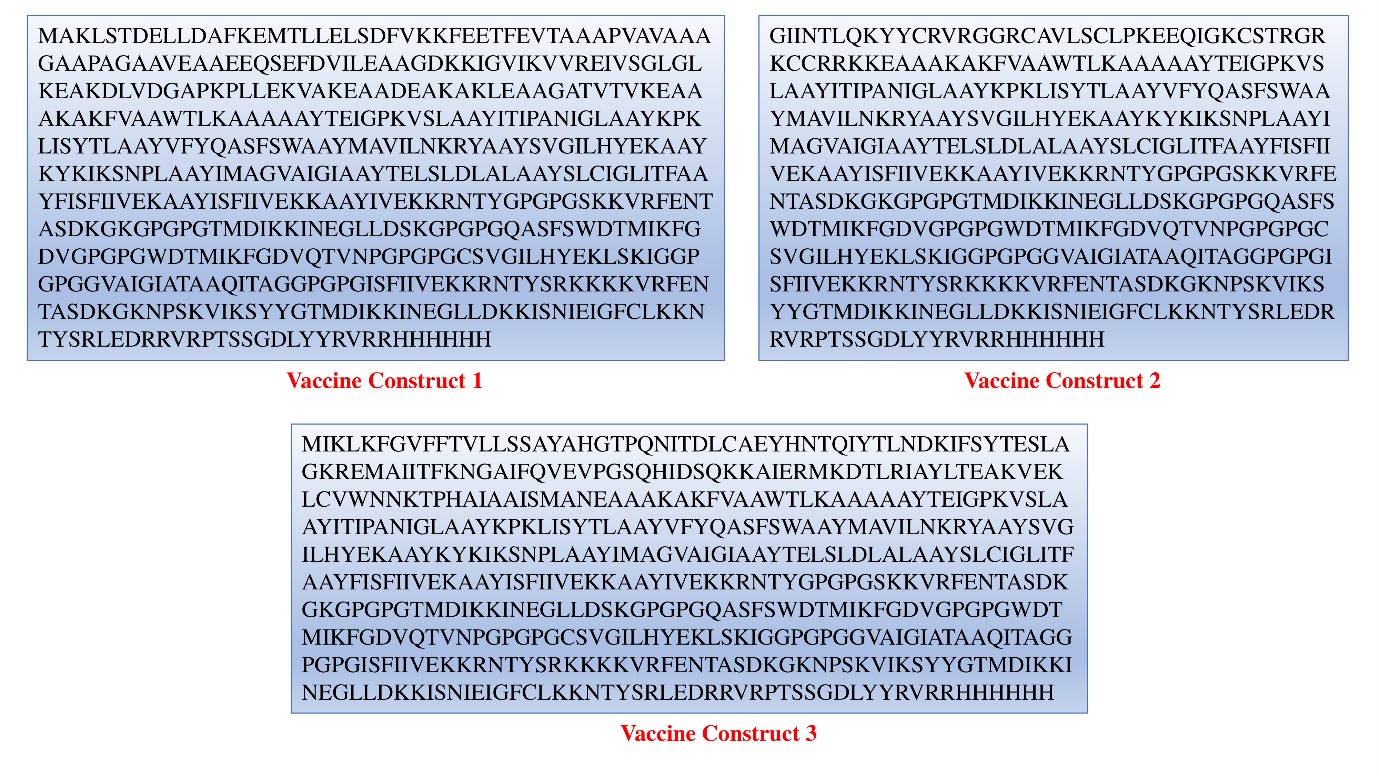
**

**Supplementary Fig.1.** Illustration of the final 3-vaccine constructed sequence. The vaccine construct 1 demonstrates the constructed sequence based on 50S ribosomal protein L7/L12, Vaccine construct 2 based on Beta defensin, and lastly vaccine construct 3 based on the CTB.
